# Effectiveness of mHealth interventions targeting physical activity, sedentary behaviour, sleep or nutrition on emotional, behavioural and eating disorders in adolescents: a systematic review and meta-analysis

**DOI:** 10.3389/fdgth.2025.1593677

**Published:** 2025-07-21

**Authors:** H. Baumann, B. Singh, A. E. Staiano, C. Gough, M. Ahmed, J. Fiedler, I. Timm, K. Wunsch, A. Button, Z. Yin, M. F. Vasiloglou, B. Sivakumar, J. M. Petersen, J. Dallinga, C. Huong, S. Schoeppe, C. L. Kracht, K. Spring, C. Maher, C. Vandelanotte

**Affiliations:** ^1^ Movement-oriented Prevention and Rehabilitation, German Sport University Cologne, Cologne, North Rhine-Westphalia, Germany; ^2^ Allied Health & Human Performance Academic Unit, University of South Australia, Adelaide, SA, Australia; ^3^ Department of Pediatric Obesity and Health Behavior, Pennington Biomedical Research Center, Baton Rouge, LA, United States; ^4^ College of Nursing and Health Sciences, Flinders University, Adelaide, SA, Australia; ^5^ Department of Nutritional Sciences and Joannah and Brian Lawson Centre for Child Nutrition, University of Toronto, Toronto, ON, Canada; ^6^ Institute of Sport and Sport Science, Karlsruhe Institute of Technology, Karlsruhe, Karlsruhe, Baden-Württemberg, Germany; ^7^ Department of Health and Social Sciences, Hochschule Fresenius, Heidelberg, Baden-Württemberg, Germany; ^8^ Department of Psychiatry, Virginia Commonwealth University, Richmond, VA, United States; ^9^ Department of Health and Kinesiology, University of Texas at San Antonio, San Antonio, TX, United States; ^10^ Independent Researcher, Bern, Switzerland; ^11^ Faculty of Health Sciences, Ontario Tech University, Oshawa, ON, Canada; ^12^ Centre of Expertise Health Innovation, The Hague University of Applied Sciences, Den Haag, South Holland, Netherlands; ^13^ Physical Activity Research Group, Appleton Institute, Central Queensland University, Rockhampton, QLD, Australia; ^14^ Division of Physical Activity and Weight Management, Department of Internal Medicine, University of Kansas Medical Centre, Kansas City, KS, United States; ^15^ School of Health and Medical Sciences, University of South Australia, Adelaide, SA, Australia

**Keywords:** mobile health, adolescent mental health, digital interventions, physical activity, sedentary behavior, nutrition, sleep

## Abstract

**Introduction:**

Mental health conditions are highly prevalent among adolescents, affecting one in seven individuals and accounting for 15% of the global disease burden in this age group. The promotion of health behaviours including physical activity, nutrition, and sleep, and reduction of sedentary behaviour, has been shown to significantly improve symptoms of mental health conditions in adolescents. However, addressing this public health challenge at a population level requires scalable interventions, such as mobile health (mHealth) interventions. However, the effectiveness of mHealth interventions in achieving clinically meaningful mental health improvements for adolescents with emotional, behavioural, or eating disorders remains unclear. Therefore, this systematic review and meta-analysis evaluated the effectiveness of mHealth behaviour change interventions aimed at improving physical activity (PA), sedentary behaviour (SB), nutrition, or sleep on outcomes related to emotional, behavioural, and eating disorders in adolescents.

**Methods:**

A systematic review and meta-analysis were conducted in accordance with PRISMA guidelines (PROSPERO ID: CRD42024591285). Eight databases were searched for randomized controlled trials (RCTs) published up to September 2024. Eligible studies included participants in early (11–14 years), middle (15–17 years) and late (18–21 years) adolescence with clinical diagnosis or self-report of emotional, behavioural, or eating disorders, where interventions targeted physical activity, sedentary behaviour, nutrition, or sleep. The cochrane risk of bias 2.0 (ROB2) and cochrane grading of recommendations assessment, development and evaluation tool (GRADE) were applied. Pooled effect sizes were calculated as standardized mean differences (SMD) with 95% confidence intervals using random-effect models.

**Results:**

Nine RCTs involving 3,703 participants were analysed across emotional, behavioural, and eating disorders. The meta-analysis yielded a significant reduction in anxiety (6 Studies, 2086 participants, SMD [95% CI] = −0.19 [−0.37, −0.01], *I*^2^ = 71%, with positive effects for sleep focussed interventions as well as multimodal interventions (PA, SB, diet, sleep) and eating disorders (3 studies, 732 participants, SMD [95% CI] = −0.23 [−0.44, −0.02], *I*^2^ = 38%, with positive effects for diet and combined diet/PA interventions). In contrast, depressive (7 Studies, 1855 participants, SMD [95%CI] of −0.12 [−0.28, −0.04], I^2^ 59%) and behavioural disorders symptoms (2 studies, 560 participants, SMD [95%CI] = −0.71 [1.77, 0.36], *I*^2^ = 95) showed no significant pooled effect. The cumulative evidence was weakened by high heterogeneity of trial design and low overall certainty of evidence as indicated by ROB2 and GRADE assessments. Across interventions, trials characterized by higher session frequency, greater intensity (e.g., more vigorous physical activity), longer duration, and hybrid delivery methods, including some face-to-face counselling were associated with larger effect sizes but reduced scalability.

**Discussion:**

These findings suggest that mHealth interventions incorporating health behavior modifications may effectively reduce anxiety and eating disorder symptoms in adolescents. However, modest and mixed effects on depression and behavioural disorders, together with a low number of included studies, considerable heterogeneity and low certainty of evidence, underscore the need for further high-quality RCTs to evaluate long-term efficacy. Combining mHealth interventions with standard clinical care may enhance symptom improvements in adolescents.

**Systematic Review Registration:**

identifier (CRD42024591285).

## Introduction

Globally, it is estimated that one in seven (15%) adolescents experiences mental health conditions (1), yet these remain largely unrecognized and untreated. The most prevalent disorders in this age group are emotional, behavioural, and eating disorders (2, 3). Emotional disorders typically include anxiety disorders (e.g., generalized anxiety disorder, panic disorder, specific phobias, and social anxiety disorder) as well as depressive disorders (e.g., major depressive disorder, dysthymia). Behavioural disorders encompass attention-deficit/hyperactivity disorder (ADHD), conduct disorders, and addiction disorders—including substance abuse (e.g., alcohol, tobacco, and cannabis) and gaming disorder. Additionally, eating disorders—particularly anorexia nervosa and bulimia nervosa—represent significant concerns. These disorders often co-occur and are associated with significant long-term consequences, including impaired academic performance, impaired social functioning, and increased risk of chronic physical and mental health conditions in adulthood (4, 5). Given their high prevalence in adolescents, these mental health conditions are the focus of this review.

Adolescents are a vulnerable population due to ongoing brain development, particularly in areas responsible for emotional regulation, impulse control, and decision-making; making them more susceptible to mental health challenges (6, 7). This period is also marked by identity formation, heightened sensitivity to peer influence, and increased exposure to digital and social media, all of which contribute to psychological distress and potential behaviours such as less healthy diets, physical inactivity, and substance use (8, 9). Many mental health conditions first emerge during adolescence (10), yet stigma, lack of awareness, and limited access to services often delay intervention (11). Additionally, adolescents differ from adults in their psychological needs, experiencing greater emotional variability and heightened sensitivity to reward and peer validation while still developing executive functioning skills (12). These factors make them more reactive to stress and external influences and create unique barriers to help-seeking, as they often rely on parents, schools (13), or digital resources for support (14). Given these developmental differences, interventions designed for adults may not be applicable, underscoring the need for age-specific, developmentally appropriate approaches (15).

Adolescence represents a developmental stage during which the formation of social and emotional habits is critical for long-term mental health. During this period, individuals typically establish sleep routines, engage in regular physical activity, and develop coping, problem-solving, interpersonal, and emotional regulation skills (1). Modifiable health behaviours, including physical activity, sedentary behaviour, nutrition, and sleep, play a pivotal role in mental health outcomes in adolescents (16, 17). For instance, physical activity has been shown to reduce symptoms of anxiety and depression, while poor sleep and unhealthy dietary patterns are associated with increased risk of emotional and behavioural disorders (18, 19). Given the widespread use of digital technologies among adolescents, mobile health (mHealth) interventions [e.g., (20)], delivered via digital technologies such as smartphone apps, wearables, and SMS, offer a promising avenue for addressing these behavioural factors in adolescents. These interventions are cost-effective, scalable, and capable of reaching diverse populations, including those in underserved or remote areas (21).

Previous systematic reviews and meta-analyses have explored the role of behavioural interventions in improving mental health outcomes, providing valuable insights but also revealing significant limitations. For example, Firth et al. (17) conducted a meta-review on the impact of behavioural psychiatry, identifying strong evidence for the benefits of physical activity, improved diet, and better sleep on mental health across various populations. Their findings indicated that physical activity had a moderate to large effect on reducing symptoms of depression and anxiety. However, the review primarily focused on adult populations and did not specifically address the unique developmental and psychological needs of adolescents. Similarly, Hoare et al. (19) systematically reviewed the associations between sedentary behaviour and mental health among adolescents, finding that excessive screen time and sedentary behaviour were linked to poorer mental health outcomes. However, their review did not explore the potential of interventions to mitigate these effects, nor did it consider the role of digital tools in addressing sedentary behaviour. Khan et al. (18) examined the therapeutic potential of physical activity for mental health, concluding that exercise interventions could significantly reduce symptoms of depression and anxiety. However, their review did not focus on adolescents or the integration of digital technologies. Furthermore, while previous reviews have contributed to understanding the role of individual health behaviours, they often lack a comprehensive approach that considers the interplay between multiple health behaviours factors (e.g., physical activity, nutrition, and sleep) or their collective impact on mental health and the interconnectedness of emotional, behavioural, and eating disorders, which often co-occur and share common underlying mechanisms.

The behavioural factors addressed by mHealth interventions—physical activity, sedentary behaviour, diet, and sleep—are all known to influence adolescent mental health through various biobehavioural pathways. Regular physical activity may reduce risk of depression and anxiety through endorphin release and reduced muscle tension, decreased inflammation and improved neuroplasticity (22). Meta-analytical evidence confirms exercise's efficacy in alleviating mild-to-moderate depressive symptoms in youth and adults. Likewise, diet quality is linked to mental health; high-fat/sugar diets correlate with poorer mood and higher stress, while nutrient-rich diets support emotional well-being (23). Nutrient deficits (e.g., omega-3, vitamins) or fluctuations in glycaemic balance can affect brain function and mood regulation. Improving diet through an intervention might therefore confer small psychological benefits—yet if done in a restrictive or weight-centric way, it could also provoke anxiety. Lastly, sleep is crucial for mental health, with poor sleep linked to elevated depression and anxiety (24). Even in clinical samples, youth with sleep disturbances show significantly higher internalizing and externalizing psychopathology. Thus, interventions that successfully improve sleep hygiene (earlier bedtimes, reduced screen use at night, etc.) can in turn reduce emotional lability and irritability.

Another gap in the literature is the limited focus on mHealth interventions, despite their growing use in health care (25). For example, Free et al. (21) demonstrated the effectiveness of mHealth interventions in improving health service delivery, however, their review did not specifically address mental health conditions nor adolescent populations. Moreover, many existing reviews (26, 27) have not systematically evaluated the effectiveness of mHealth interventions targeting multiple health behaviours in improving mental health outcomes. This represents a significant oversight, given the potential of digital tools to deliver integrated, personalized, and scalable interventions that address the complex interplay between health behaviours and mental health.

This systematic review and meta-analysis aimed to address these gaps by evaluating the effectiveness of mHealth interventions targeting physical activity, sedentary behaviour, nutrition, or sleep on emotional, behavioural, and eating disorders exclusively in adolescents aged 11–21 years. Additionally, this review explored the mechanisms behind health behaviour change interventions with more than one target behaviour, offering insights into how mHealth interventions can be optimized to address the unique mental health challenges faced by adolescents. The findings will inform the development of targeted, evidence-based mHealth strategies to address the interconnected challenges of mental health and health behaviours in adolescents, ultimately contributing to improved well-being and reduced disease burden in this critical age group.

## Methods

### Study design

This systematic review and meta-analysis were conducted in accordance with the 2020 PRISMA (Preferred Reporting Items for Systematic Reviews and Meta-Analyses) guidelines. The protocol for this review was prospectively registered in PROSPERO (Registration No.: CRD42024591285). The focus was on randomized controlled trials (RCTs) evaluating mHealth interventions that target physical activity, sedentary behavior, nutrition, or sleep in adolescents, with emotional, behavioural, and eating disorders as the primary outcomes.

### Eligibility criteria

Eligibility criteria were defined using the PICOS framework (Population, Intervention, Comparator, Outcomes, and Study Design) (28). The target population included adolescents aged 11–21 years with emotional, behavioural, or eating disorders, either diagnosed or self-reported. Eligible interventions were digital behavioural mHealth interventions delivered via mobile applications, wearable devices, or SMS-based communication, aimed at improving physical activity, sedentary behaviour, nutrition, or sleep. “Eligible studies were required to deliver the main behaviour-change content predominantly (≥75% of contact time) via digital technologies (e.g., apps, web platforms, SMS). Ancillary non-digital elements—such as a one-off orientation session, brief therapist feedback, or parent-directed materials—were permitted provided they remained clearly secondary to the digital component.” Control groups included no intervention, active controls, treatment as usual, waitlist controls, or attention-matched sham interventions. Physical activity, sedentary behaviour, diet or sleep interventions delivered via traditional methods (e.g., face to face) as a comparison to digital interventions were not eligible to be included. Primary outcomes were changes in emotional disorders (e.g., depression, anxiety), behavioural disorders (e.g., ADHD, conduct disorders, addiction), and eating disorders (e.g., anorexia nervosa, bulimia nervosa) measured at baseline and post-intervention. Only studies reporting validated outcome measures were eligible (e.g., qualitative assessments were excluded). Inclusion was limited to RCTs and it's derivations (e.g., cluster RCTs, waitlist-controlled trials, and quasi-RCT's). Observational studies, case reports, prevalence studies, and theoretical papers were excluded. No language or publication date restrictions were applied.

### Information sources and search strategy

A systematic search was conducted across PubMed, MEDLINE, EMBASE, PsycINFO, the Cochrane Central Register of Controlled Trials, EMCARE, PEDro, and Web of Science. Search terms were developed based on Medical Subject Headings and free-text keywords related to mHealth, physical activity, sedentary behaviour, nutrition, sleep, and mental health outcomes. The final search was completed on 20th September 2024 and imported into Covidence Software (29). All references were automatically screened for duplicates. The full search strategy is attached as Supplementary Material.

### Study selection

**Figure 1:**
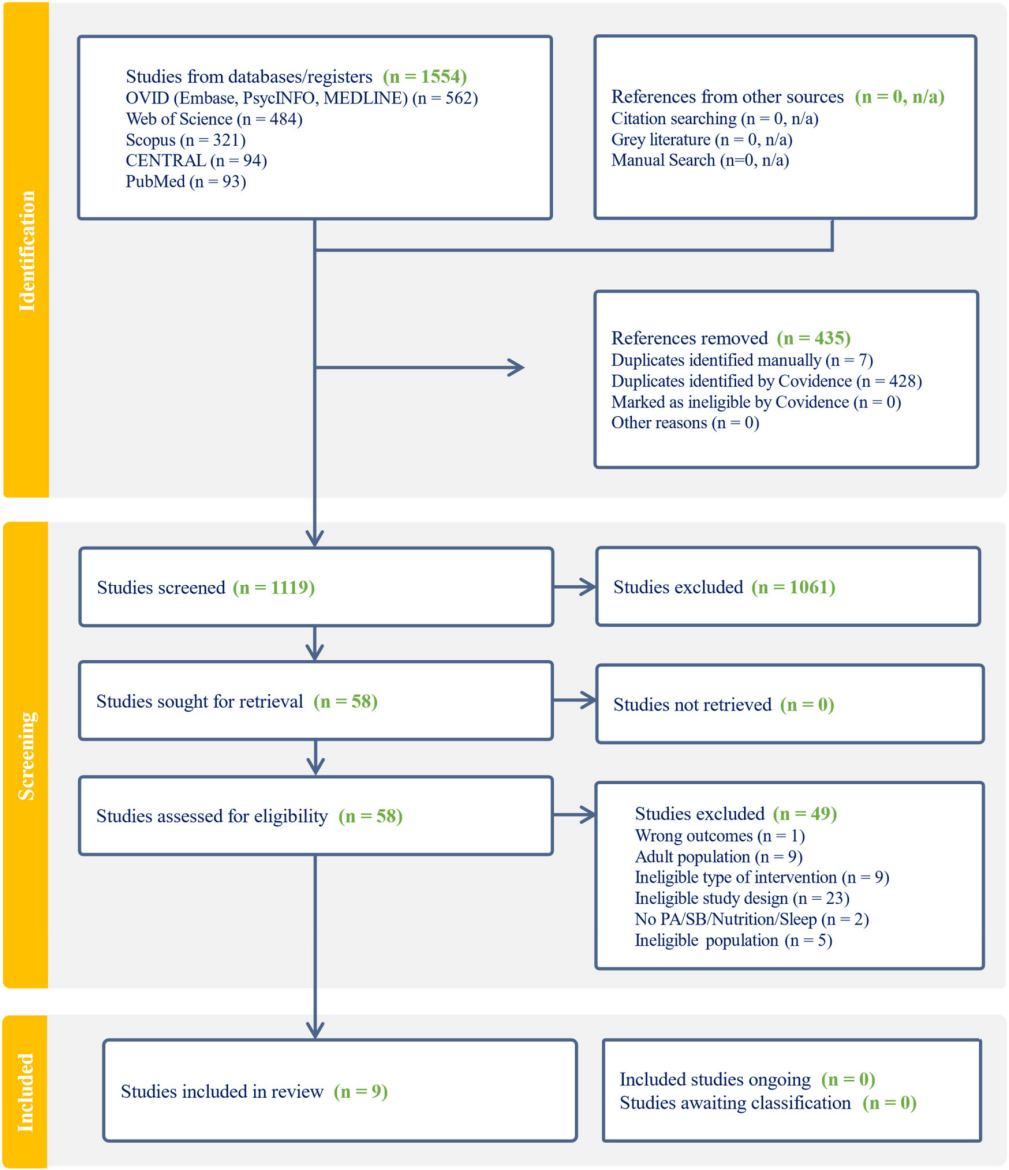
PRISMA flow chart.

The selection process followed a multi-phase approach, with all identified records first imported into Zotero (30) for reference management and then to Covidence software for review management, where duplicates were automatically removed. Title and abstracts were screened in duplicate by all authors based on predefined eligibility criteria, resolving discrepancies through discussion with HB and BS. Full-text articles were retrieved for all potentially eligible studies and independently screened in duplicate, in pairs by 17 independent reviewers (HB, CG, AS, KW, MA, JF, CK, JP, MV, BS, CV, JD, IT, SS, KS, KS, and AB), with disagreements resolved by HB and BS. The PRISMA flowchart (Figure 1) outlines the selection process, including exclusion reasons at each stage. The interrater reliability for all stages of the screening process was assessed using Cohen's Kappa.

### Data collection and extraction

Data extraction was performed using a standardized and piloted data extraction template and quality assessment template in Covidence to ensure consistency and reliability. Nine reviewers (HB, ZY, JF, CG, AS, BS, KW, SS, and MA) independently extracted data from the included studies, in duplicate. Discrepancies were resolved through consensus or, if needed, consultation with a third reviewer. Extracted data included study identification details, methodological characteristics, population characteristics, intervention and control conditions, sample demographics, and relevant mental health outcomes. Study identification covered authorship, institutional affiliation, sponsorship, country, and study setting. Methodological characteristics included study design and group allocation. Population characteristics encompassed inclusion and exclusion criteria, baseline group differences, total sample size, dropout rates, disorder classification, and socioeconomic status. Data on intervention and control conditions included participant allocation, duration, frequency, targeted health behaviours, type of behaviour targeting, and delivery format. Sample demographics comprised mean age, age range, gender distribution, and age group classification. Mental health outcomes were extracted for both intervention and control groups, including outcome measures, mean scores, confidence intervals, and standard deviations across time points. Where data were incomplete, unclear or missing, study authors were contacted for clarification. For studies with multiple outcome measures, the most relevant measure for each targeted disorder was selected following pre-defined criteria, prioritizing validated and commonly used scales.

### Risk of bias assessment

The risk of bias in individual studies was assessed using the Cochrane Risk of Bias 2 (ROB2) tool (31), which evaluates five domains: bias arising from the randomization process (*n* = 3 items), bias due to deviations from intended interventions (*n* = 7 items), bias due to missing outcome data (*n* = 4 items), bias in outcome measurement (*n* = 5 items), and bias in the selection of the reported result (*n* = 3 items), as well as an overall bias assessment derived from these domains. Nine reviewers (HB, ZY, JF, CG, AS, BS, KW, SS, and MA) independently evaluated the risk of bias for each study in duplicate, with a third reviewer (HB) consulted to resolve any discrepancies. The ROB2 tool provides predefined criteria for evaluating methodological rigor in each domain, ensuring a standardized assessment of study quality. Reviewers were required to apply these criteria systematically and substantiate their judgments with specific textual evidence from the studies. The resulting assessments were integrated into the overall synthesis to evaluate the quality and reliability of the evidence base. The certainty of evidence was further evaluated by using the Cochrane Grading of Recommendations Assessment, Development and Evaluation Tool (GRADE approach) (32).

### Summary measures and effect size calculation

The primary summary measure for all outcomes was the standardized mean difference (SMD) with 95% confidence intervals (CIs), selected to ensure comparability across studies utilizing different measurement instruments. To compute effect sizes, sample sizes, means, and standard deviations were extracted for both baseline and post-intervention time points. In cases where multiple measures were reported for the same outcome, the most clinically relevant and methodologically valid measure was prioritized. If a study provided standard errors instead of standard deviations, the latter was derived using the RevMan Web calculator (33) to ensure consistency in effect size estimation.

### Data synthesis and statistical analysis

A random-effects meta-analysis was conducted using Revman (33) and the RStudio Metaphor package (34) to account for heterogeneity across studies. The standardized mean difference (SMD) was used as the primary effect measure, ensuring comparability across studies utilizing different measurement tools. Statistical heterogeneity was assessed using the I^2^ statistic, with values exceeding 75% indicating considerable heterogeneity, values between 50% and 75% suggesting substantial heterogeneity, and values below 40% reflecting limited heterogeneity. The Restricted Maximum-Likelihood (REML) method was employed as the heterogeneity estimator to provide an unbiased variance estimate. Confidence intervals for the summary effect were calculated using the Wald-type method. To evaluate potential publication bias, contour-enhanced funnel plots, Egger's test, and Begg's test were applied. Where publication bias was detected, adjustments were made using Duval and Tweedie's trim-and-fill method.

### Ethics and further analysis

Although the study protocol registered in PROSPERO initially planned for subgroup analyses based on age group (early adolescence: 11–14 years, middle adolescence: 15–17 years, late adolescence: 18–21 years), intervention type (mobile application, wearable device, SMS-based intervention), targeted health behaviour (physical activity, sedentary behaviour, nutrition, or sleep), and risk of bias level, these analyses could not be conducted due to the limited number of included studies. Sensitivity analyses were performed by conducting a leave-one-out analysis, systematically omitting each study one at a time to evaluate the robustness of the overall findings and identify any studies with a disproportionate influence on the pooled effect size. As this study synthesized data from previously published research, ethical approval was not required.

## Results

### Study selection

Of the 1,554 records identified, nine studies met the eligibility criteria (Figure 1). For the title and abstract (TA) screening, the kappa coefficient (range: −1 = complete disagreement to +1 = perfect agreement), was *κ* = 0.299, indicating fair agreement between reviewers. Similarly, the full-text screening yielded a kappa coefficient of *κ* = 0.374, also reflecting fair agreement. Moderate discrepancies were observed in both stages, but these were resolved per protocol.

### Study characteristics

An overview of all study characteristics is shown in Table 1. At the study level, eight of the included studies (88%) (35–42) were conducted in countries with a human development index >0.9, with an even distribution across early adolescence (11–14 years, 44%, *n* = 4) and late adolescence (18–21 years, 44%, *n* = 4), and only one study (40) focusing on middle adolescence (15–17 years, 11%, *n* = 1) and also being the largest study with *n* = 1,349 participants. The mean age across studies was 15.93 ± 2.11 years. Female participants accounted for 71% (*n* = 2,297) of the total sample, with two studies exclusively targeting female populations (37, 38). Randomized controlled trials (RCTs) comprised 33% (*n* = 3) of the studies, with the remainder consisting of cluster RCTs (33%, *n* = 3), waitlist-controlled trials (22%, *n* = 2), and quasi-RCTs (11%, *n* = 1). The studies collectively included a total of 3,219 participants with overall sample sizes considerably varying from *n* = 100 up to *n* = 1,349, with a mean of 178 ± 202 participants per study arm. Nearly two-thirds of the total sample was provided by the study of Fitzsimmons-Craft et al. (37) (*n* = 690) and Lahtinen et al. (40) (*n* = 1,349). The dropout rate averaged 27% (*n* = 869), with the highest dropout rates reported in the studies of Egilsson et al. (36) (63%, *n* = 147), Werner-Seidel et al. (42) (44%, *n* = 74), and Lahtinen et al. (40) (42%, *n* = 284). Studies using app-only interventions reported higher dropout rates compared to those employing hybrid formats. For example, the study by Egilsson et al. (36), focusing on younger adolescents (13.5 ± 0.63 years) had the highest dropout rate at 63% (*n* = 147), while the study of Anastasiadou et al. (2020), using a combined face-to-face and app approaches, had a lower dropout rate of 37% (*n* = 53).

**Table 1:** Descriptive characteristics of included studies across study, intervention, and outcome level.

Study level				Intervention level								Outcome level		
Author (Year)	Country (Population)	Age group (Mean Age ± SD)	Total N, % female, (% dropout)	Design	Targeted health behaviour	Intervention vs. control	*N*	FITT-principles: Frequency (Sessions/week), Intensity (Rating), Time (duration in weeks) and Type (delivery)				Disorder group	Severity of observed specific disorder(s)	Relevant Outcomes: SMD (Measurement Scale)
								F	I	T	T			
Anastasiadou et al. (35)	Spain (Adolescent mental Health services)	Late (17.25 ± 3.54)	106 91 (37)	RCT	diet	CBT + TC-App	53	7	High	12	FTF + App	Eating	Moderate to severe eating or feeding disorder	Depression: 0.01 (BDI-II) Anxiety: 0.14 (STAI) Eating disorder: −0.07 (EDE-Q)
						CBT	53	1	Moderate	12	FTF			
Egilsson et al. (36)	Iceland (Students in public Schools)	Early (13.50 ± 0.63)	223 47 (63)	Cluster RCT	PA, SB, diet, sleep	SidekickHealth	117	7	Moderate	6	App	Emotional	Mild to severe anxiety or depression	Depression: 0.16 (RCADS) Anxiety: −0.5 (RCADS)
						TAU	106	7	Moderate	6	App			
Fitzsimmons-Craft et al. (37)	United States (female students)	Late (21.63 ± 4.19)	690 100 (17)	Cluster RCT	diet	SB-ED	385	1	High	32	Web + App	Eating/Behavioural	Moderate to severe Bulimia nervosa or binge-eating	Anxiety: −0.16 (PROMIS) Eating disorder: −0.36 (EDE-Q) Behavioral Disorder −0.18 (F)
						TAU	305	SP	Low	32	Diary			
Hahn et al. (38)	United States (Overweight woman)	Late (20.4 ± 3.1)	200 100 (6)	RCT	PA, diet	MyFitnessPal	100	7	Low	12	App	Eating	Mild unspecified eating disorder	Depression: −0.02 (CESD-R) Anxiety: 0.13 (STAI) Eating disorder: −0.10 (EDE-Q)
						No intervention	100	0	None	12	None			
Kauer et al. (39)	Australia (General health practices)	Late (18.0 ± 3.2)	114 78 (23)	Cluster RCT	PA, diet, sleep	Mobiletype	68	14	Moderate	2-4	App	Emotional	Moderate depression	Depression: −0.01 (PHQ-9)
						Attention control	46	14	Low	2-4	App			
Lahtinen and Salmivalli (40)	Finland (Secondary education)	Middle (16.8 ± 0.8)	1349 73 (42)	Waitlist RCT	Sleep	MBP	676	1	Moderate	8	Web	Emotional	Mild anxiety or depression	Depression: −0.16 (PHQ-9), Anxiety: −0.25 (GAD-7)
						Waitlist	673	0	None	8	None			
Peuters et al. (41)	Canada (secondary education)	Early (13.63 ± 0.96)	279 62 (3)	Quasi RCT	PA, SB, diet, sleep	LIFEGOALS school	184	SP	Moderate	12	FTF + App	Emotional	Mild depression	Depression: −0.39 (PROMIS)
						LIFEGOALS remote	95	SP	Moderate	12	App			
Werner-Seidel et al. (42, 43)	Australia (Students in Secondary education)	Early (14.74 ± 1.27)	168 70 (44)	RCT	Sleep	Sleep Ninja	85	7	High	6	APP	Emotional	Moderate depression and insomnia	Depression: −0.37 (PHQ-9), Anxiety: −0.29 (GAD-7)
						Text Message	83	1	Low	6	SMS			
Zhao et al. (44)	China (patients from hospital)	Early (8.4 ± 1.3)	90 21 (11)	Waitlist RCT	PA	Brainfit	44	3	High	4	AR-App	Behavioral	Mild to severe ADHD	ADHD: −1.27 (SNAP-IV)
						Waitlist	46	0	None	4	None			
Summary	EUR: 33% (3) AMR: 33% (3) WPR: 33% (3)	Early: 44% (4) Middle: 11% (1) Late: 44% (4)	Total N: 3219 71% Female 27% Dropout	RCT: 33% (3) Cluster: 33% (3) Waitlist: 22% (2) Quasi: 11% (1)	PA 55% (5) SB 22% (2) Diet 66% (6) Sleep 55% (5)	89% active control	M:178 SD:202	M:4.6 SD:4.8	Low: 22% (4) Moderate: 39% (7) High: 22% (4) None: 17% (3)	M: 10.5 SD: 8.5	App: 61% (11) Web: 11% (2) SMS: 5% (1) FTF: 17% (3) Diary: 5% (1) None: 17% (3)	Em: 55% (5) Ea: 33% (3) Be: 11% (1)	Mild: 33% (5) Moderate: 40% (6) Severe: 27% (4)	PHQ-9: 16% (3) EDE-Q: 16% (3) STAI: 11% (2) PROMIS: 11% (2) GAD-7: 11% (2) RCADS: 11% (2)

Abbreviations: EUR, European region; AMR, Americas region; WPR, western-pacific region; RCT, randomized controlled trial; PA, physical activity; SB, sedentary behaviour; CBT, cognitive behavioural therapy; TAU, treatment as usual; MBP, mindfulness based program; FTF, face-to-face; AR, augmented reality; Em, emotional; Ea, eating; Be, behavioral; ADHD, attention deficit hyperactivity disorder; PHQ-9, patient health questionnaire-9; EDE-Q, eating disorder examination-questionnaire; STAI, state-trait-anxiety inventory; PROMIS, patient-reported outcomes measurement information system; GAD-7, generalized anxiety disorder scale-7; RCADS, revised children's anxiety and depression scale; BDI-II, beck-depressions-inventory revision; RCADS, revised children's anxiety and depression scale; CESD-R, centre for epidemiologic studies depression scale; SNAP-IV, teacher and parent ADHD Rating Scale from Swanson, Nolan, and Pelham; F, frequency.

At intervention level, diet was the most targeted health behaviour across studies (66%, *n* = 6), followed by physical activity (55%, *n* = 5) and sleep (55%, *n* = 5), while sedentary behaviour was targeted in 22% (*n* = 2) of studies (*n* = 4 studies targeted multiple health behaviours at once). Multifactorial interventions, such as SidekickHealth (36) and LIFEGOALS (41), addressed multiple health behaviours, often applied to mild mental health conditions (see Table 1). Conversely, interventions targeting more severe conditions, such as those by Anastasiadou et al. (35) and Lahtinen et al. (40), typically only focused on one health behaviour. Interventions for moderate to severe disorders were associated with higher session frequency and intensity (see FITT principles in Table 1) and higher app engagement related to the targeted health behaviour. As presented in Table 1, studies with multi-behavior approaches, higher session frequencies, longer duration, hybrid delivery formats show improved adherence and more robust outcomes. For instance, in their study, Anastasiadou et al. (35) implemented seven sessions per week with high intensity diet related CBT + TC App sessions for moderate to severe cases of eating disorders. In Contrast, Zhao et al. (44) applied three sessions per week with high intensity physical activity for mild to severe forms of ADHD.

In contrast, interventions for milder mental health cases often featured lower frequencies or self-paced formats, such as in the study by Fitzsimmons-Craft et al. (37), which used a self-paced approach for bulimia nervosa and binge-eating disorders. Intervention durations varied widely, averaging 10.5 ± 8.5 weeks. The study by Fitzsimmons-Craft et al. (37) reported the longest intervention period of 32 weeks, while shorter durations were observed in the studies by Zhao et al. (44) (4 weeks) and Werner-Seidel et al. (42) (6 weeks). Delivery approaches for both intervention and control groups (there was a total of 18 study arms across the 9 included studies) were predominantly app-based (61%, *n* = 11 study arms), with additional approaches also including web (11%, *n* = 2 study arms), face-to-face (17%, *n* = 3 studyarms), augmented reality (5%, *n* = 1 studyarm), and diary-based interventions (5%, *n* = 1 studyarm).

At the outcome level, emotional disorders were the most frequently addressed disorder group (55%, *n* = 5), followed by eating disorders (33%, *n* = 3) and behavioural disorders (11%, *n* = 2). Disorder severity was predominantly moderate (40%, *n* = 6), with fewer studies addressing mild (33%, *n* = 5) or severe (27%, *n* = 4) conditions. Emotional disorders were predominantly assessed using validated psychometric instruments, with the Patient Health Questionnaire-9 (PHQ-9) applied in 3 of the 5 studies focused on emotional disorders and the Generalized Anxiety Disorder-7 (GAD-7) in 2 of these 5 studies. Eating disorders were evaluated using the Eating Disorder Examination Questionnaire (EDE-Q) in all 3 of the eating disorder of studies. Behavioural disorders, including ADHD, were with the Swanson, Nolan, and Pelham-IV Scale (SNAP-IV) (44) or by frequency measures of specific behaviours (37). Across all categories, standardized assessment tools were consistently employed to ensure methodological rigor and comparability across studies. The majority of studies targeting eating disorders, such as the studies by Fitzsimmons-Craft et al. (37) and Hahn et al. (38), exclusively included female participants. In contrast, studies addressing emotional disorders, such as Egilsson et al. (36), had more balanced gender distributions (47% female, *n* = 147). Studies targeting severe disorders with behavioural mHealth interventions demonstrated lower effects sizes then Studies targeting mild to moderate disorder symptoms.

### Risk of bias

**Figure 2:**
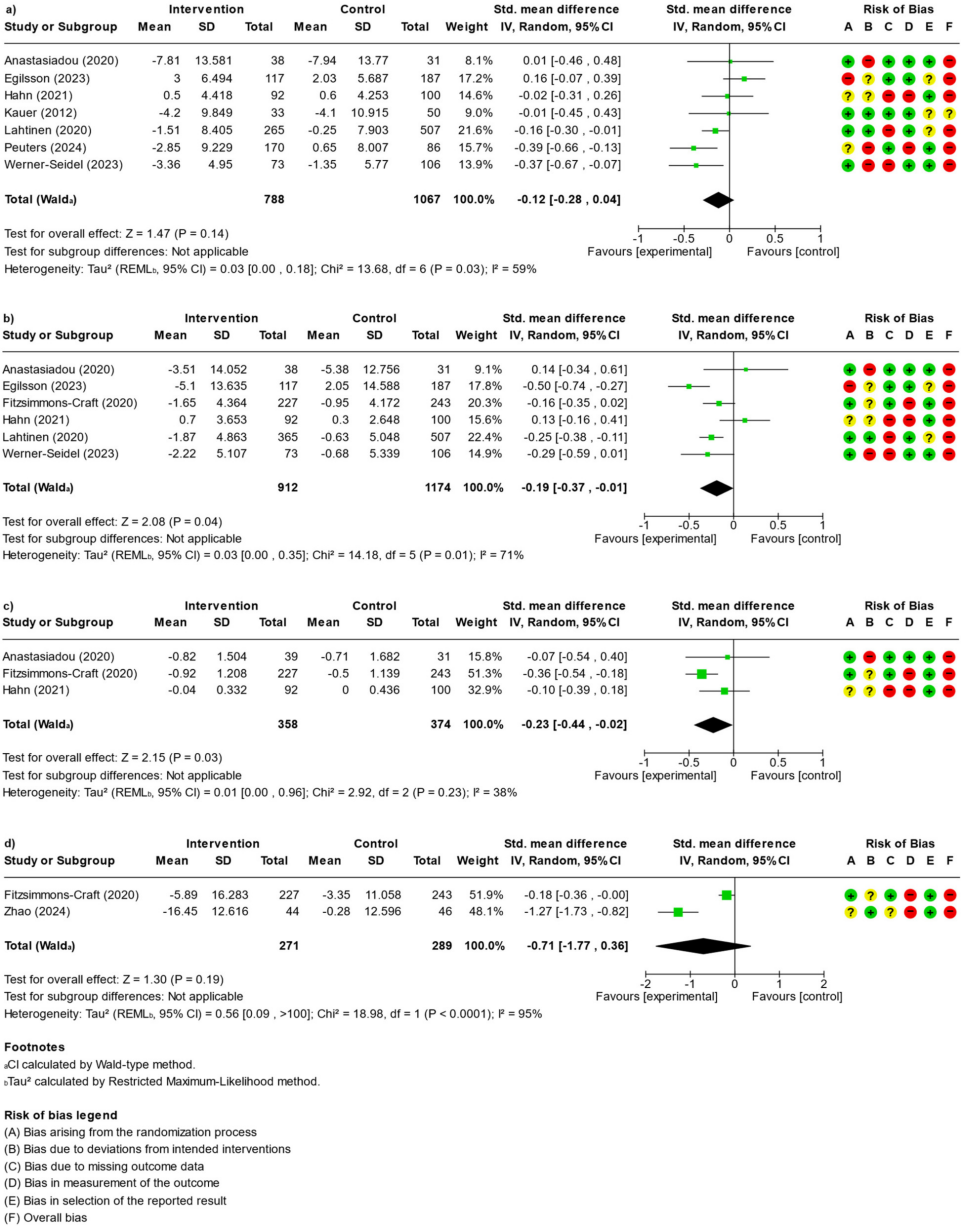
Forest plots and risk of bias assessment for **(a)** depression, **(b)** anxiety, **(c)** eating disorders and **(d)** behavioural disorder outcomes (“+” = low risk of bias, “?” = unclear risk of bias), “−” = high risk of bias).

Across the 9 studies, 11 out of 45 ratings (5 dimensions × 9 studies) indicated high risk of bias, and 10 ratings showed an unclear risk of bias, resulting in an overall rating of 1 (11%) study with unclear risk of bias and 8 (88%) studies with a high risk of bias. Potential biases frequently occurred in dimensions B (bias due to deviations from intended interventions), C (bias due to missing outcome data) and D (bias in the measurement of the outcome). More detailed ROB information for each study can be found in the forest plots for the meta-analysis (see Figure 2).

### Synthesis of results

Although the data from nine studies were insufficient to conduct any subgroup analyses, there was adequate information available to perform meta-analyses on emotional disorders, including depression and anxiety, as well as eating disorders and behavioral disorders (see Figure 2). Heterogeneity was lowest for eating disorders and highest for behavioural disorders. Study contributions to the pooled effects varied from 8% to 51%. The forest plots in Figure 2 indicate significant reduction in anxiety [6 Studies, 2,086 participants, SMD (95%CI) = −0.19 (−0.37, −0.01), *I*^2^ = 71%] with positive effects for sleep focussed interventions as well as multimodal interventions (PA, SB, diet, sleep) particularly effective interventions being the SidekickHealth App (36) and the Sleep Ninja App (42), SB-ED-App (37) and the MBP-Webapp (40). Eating disorders [3 studies, 732 participants, SMD (95%CI) = −0.23 (−0.44, −0.02), I^2^ = 38%] also showed significant results with positive effects for diet and combined diet/PA interventions and effective interventions being the TC-App used by Anastasiadou et al. (35), SB-ED-App used by Fitzsimmons-Craft et al. (37) and the MyFitnessPal-App used by Hahn et al. (38).

**Figure 3:**
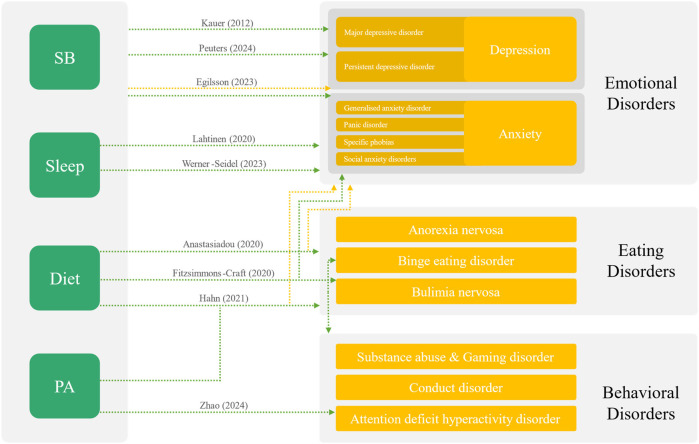
Overview of the studies categorized by targeted health behaviour and primary mental health outcomes as characterized by protocol (arrows indicate if the meta-analysis results showed a symptom reduction (green arrow) or symptom exacerbation (orange arrow).

In contrast, depressive [7 studies, 1,855 participants, SMD (95%CI) of −0.12 (−0.28, 0.04), *I*^2^ 59%] and behavioural disorders symptoms [2 studies, 560 participants, SMD (95%CI) = −0.71 (−1.77, 0.36), *I*^2^ = 95] showed no significant pooled effect. To illustrate findings, Figure 3 further depicts these relationships by mapping the targeted health behaviours to the corresponding mental health outcomes, highlighting the heterogeneity in intervention target behaviours and outcome measures across studies. It presents an overview of the included studies categorized by their targeted health behaviour and primary mental health outcomes and indicates, if the meta-analysis results showed a symptom reduction (green arrow) or symptom exacerbation (orange arrow). The diagram illustrates the complexity of the relationships within the integrated studies by mapping specific mHealth intervention target behaviours to the respective mental health outcomes. The origin and termination points of each arrow were chosen intentionally to convey two pieces of information: (i) the health behaviour(s) targeted by the intervention and (ii) the mental-health outcome(s) evaluated in the study. Grey frames aggregate related constructs in order to visualise the degree of specificity or breadth with which individual studies addressed behaviours and outcomes. (i) Intervention side (left): When an arrow originates at the boundary of the grey frame that encloses all four health behaviours [sedentary behaviour (SB), sleep, diet and physical activity (PA)], the corresponding intervention targeted all four behaviours [e.g., (41)]. If the arrow begins at one specific behaviour (e.g., PA), the intervention focused exclusively on that behaviour [e.g., (44)]. (ii) Outcome side (right): Arrows that terminate at the outer margin of a grey frame (e.g., “Emotional Disorders”) indicate that the study reported outcomes at the category level, without differentiating between individual disorders [e.g., (20)]. Arrows that end within a specific disorder box (e.g., “Attention Deficit Hyperactivity Disorder”) signal that outcomes were assessed for that particular diagnosis only, as illustrated by Zhao et al. (44).

The figure highlights the variability in intervention approaches, with some studies employing multimodal strategies, integrating multiple health behaviours (36, 39, 41), while others focus on a single behavioural target (35, 37, 40, 43, 44). This differentiation is visually represented by the origin and termination points of the arrows, indicating the specific behaviours addressed and their corresponding mental health outcomes. A similar pattern is observed in the scope of the targeted outcomes. While some studies address broad categories of mental health conditions, such as emotional disorders (36, 40), others focus on specific disorders, such as ADHD (44). Furthermore, some studies target multiple specific and grouped mental health outcomes simultaneously (37), demonstrating the heterogeneity of the evidence base.

### Reporting bias

**Figure 4:**
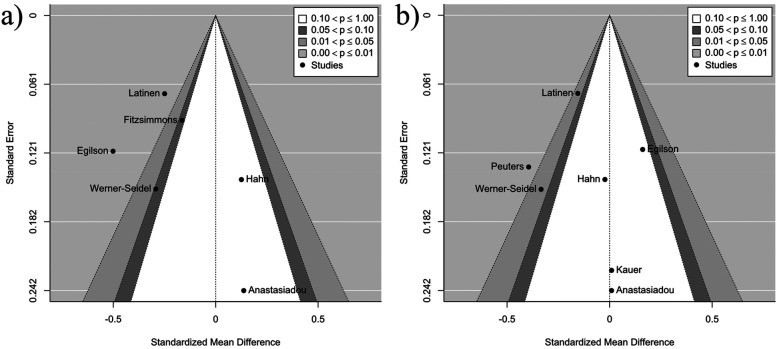
Contour-enhanced funnel plot for **(a)** depression, **(b)** anxiety outcomes.

Contour-enhanced funnel plots (Figure 4) provide insights into publication bias and heterogeneity across anxiety and depression outcomes. For behavioural and eating disorder outcomes, the number of studies included was too small (*n* ≤ 6) to perform a funnel plot analysis. For depression outcomes, the plot showed symmetry, indicating minimal publication bias. The removal of one influential study reduced heterogeneity from 52.82% to 14.76%, suggesting that the results are reliable with minimal variability. In contrast, anxiety outcomes revealed slight asymmetry, pointing to moderate publication bias. Behavioural disorders, with only two studies available, provided limited data for analysis. For eating disorders, the pooled effect size (−0.40) suggested a moderate reduction in symptoms, but the scarcity of data weakened the robustness of the findings.

Across all outcome categories, depression and anxiety outcomes demonstrated minimal bias and manageable heterogeneity, while eating disorder results showed greater variability and sensitivity to outliers. Behavioural disorder findings were constrained by insufficient data. An additional leave-one-out analysis revealed varying degrees of influence by individual studies across outcomes. For behavioural outcomes, the analysis was limited due to the small number of studies (*n* = 2), making meaningful interpretations of influence of heterogeneity impossible. For depressive symptoms, the pooled effect size ranged from −0.18 to −0.07, and one study significantly influenced the results (*p* = 0.002), reducing heterogeneity from *I*^2^ = 52.82 percent to *I*^2^ = 14.76 percent when excluded. For eating disorders, the pooled effect size ranged from −0.30 to −0.09, with two studies showing significant influence (*p* = 0.01 and *p* = 0.043); removing these studies changed heterogeneity from *I*^2^ = 24.98 percent to *I*^2^ = 20.23 percent and *I*^2^ = 54.70 percent, respectively. For anxiety symptoms, the pooled effect size ranged from −0.26 to −0.14, with two studies significantly affecting the results (*p* = 0.0003 and *p* = 0.016). Removing one study reduced heterogeneity from *I*^2^ = 65.56 percent to *I*^2^ = 45.61 percent, while removing another increased it to *I*^2^ = 72.09 percent. Overall, while the results are generally stable, specific studies had a disproportionate impact on the pooled effect sizes and heterogeneity, particularly for depressive symptoms, eating disorders, and anxiety symptoms, underlining the importance of study-level characteristics and their role in driving heterogeneity.

### Certainty of evidence

The overall appraisal using the Cochrane GRADE framework (45, 46) reveals substantial methodological challenges across the evidence base (see Table 2). In broad terms, the certainty of the findings is largely compromised by serious issues in study design and reporting—most notably, risk of bias and imprecision consistently affected the reliability of the effect estimates. Although a greater number of studies underpin the depression and anxiety outcomes, these were nonetheless downgraded on multiple fronts, resulting in low confidence regarding the observed effects. For eating disorders, the evidence, albeit based on fewer studies, demonstrated somewhat greater consistency and precision, which translated into a moderate level of certainty. In contrast, the very limited evidence available for behavioural disorders was marked by pronounced inconsistency and a strong suspicion of publication bias, leading to an overall classification of very low certainty.

**Table 2:** Summary of findings, based on GRADE approach.

Disorder group	No. of studies	Study design	Risk of bias	Inconsistency	Indirectness	Imprecision	Publication bias	Relative Risk (95% CI)	Certainty
Depression	7	RCT	Serious (−1)	Serious (−1)	Not Serious (0)	Serious (−1)	Undetected (0)	0.12 (−0.28/0.04)	Low
Anxiety	6	RCT	Serious (−1)	Serious (−1)	Not Serious (0)	Serious (−1)	Undetected (0)	−0.19 (−0.37/-0.01)	Low
Eating	3	RCT	Serious (−1)	Not Serious (0)	Not Serious (0)	Serious (−1)	Undetected (0)	−0.34 (−0.44/−0.02)	Moderate
Behavioural	2	RCT	Serious -(1)	Very Serious (−2)	Not Serious (0)	Serious (−1)	Suspected (−1)	−0.71 (−1.77/0.36)	Very Low

RCT, randomized controlled trial.

## Discussion

This systematic review and meta-analysis evaluated the effectiveness of mHealth interventions targeting physical activity, sedentary behaviour, nutrition, or sleep on emotional, behavioural and eating disorders in adolescents aged 11–21 years. It identified nine randomized controlled trials targeting early, middle and late adolescents. Despite the heterogeneity of included studies, the meta-analysis showed significant small reductions in anxiety and eating disorders but non-significant trends for reductions in depression and behavioural disorders.

### Anxiety

Our review found that mHealth interventions targeting behaviours like physical activity, diet, and sleep can lead to reductions in anxiety symptoms among adolescents, even when formal psychotherapy is not provided (see Table 1). Positive effects were found in sleep focussed interventions as well as multimodal interventions (PA, SB, diet, sleep) particularly effective interventions being the SidekickHealth App (36) and the Sleep Ninja App (42), SB-ED-App (37) and the MBP-Webapp (40). This aligns with prior research showing that both digital cognitive-behavioural therapy (CBT) programs and health behaviour interventions can—especially in mild conditions—effectively reduce anxiety in adolescents, as demonstrated in a recent meta-analysis (47). Blending online modules with therapist support (a “hybrid” model) could also harness the convenience of technology and the personal connection of face-to-face therapy (48), but would also limit scalability. Of course, this does not diminish the value of therapy for more severe or complex anxiety disorders, but it underscores the potential of accessible behavioural modifications as an early-line or adjunct strategy. Several trials in this review that used a combination of sleep and physical activity interventions (40, 42) have reported meaningful reductions in adolescent anxiety symptoms which aligns with those reported in previous systematic reviews in adults (18, 49, 50). Improvements in anxiety symptoms were noted across various studies, even when mHealth interventions focused on all behavioural outcomes simultaneously (physical activity, sedentary behaviour, diet and sleep [e.g., (36)] rather than including standard psychological therapies like cognitive behavioural therapy (51). This aligns with broader evidence that behavioural adaptation (e.g., regular exercise) and healthy routines exert anxiolytic effects in young populations. A systematic review concluded that physical activity effectively reduces anxiety in adolescents and young adults, with active participants experiencing greater symptom reduction than inactive controls (52). Potential mechanisms include improved autonomic regulation, changes in neurotransmitter levels, anti-inflammatory effects, and increased neurotrophic factors (53). mHealth interventions also often improve sleep quality, which can break the vicious cycle between insomnia and anxiety (54). Better sleep and fitness contribute to improved emotion regulation and reduced anxiety (52). While these behavioural changes help, they may not fully resolve symptoms in all adolescents, particularly those with severe anxiety. A stepped-care approach—starting with mHealth tools in mild conditions and progressing to therapy in more severe cases if needed—may be most effective: beginning with behavioural mHealth tools to capitalize on adolescents’ receptivity to tech-based coaching and then stepping up to blended or face-to-face therapy if needed for those who do not respond sufficiently.

### Eating disorders

The meta-analytic results on eating disorders suggest that well-designed digital tools—especially those integrating parental support or professional guidance—can reduce behaviours like binge eating, restrictive dieting, or unhealthy weight control in young people. Positive effects were found for diet only and combined diet/physical activity interventions, with particularly effective interventions being the TC-App used by Anastasiadou et al. (35), the SB-ED-App used by Fitzsimmons-Craft et al. (37) and the MyFitnessPal-App used by Hahn et al. (38). However, focusing too narrowly or intensively on eating behaviour in an intervention can, in some cases, heighten anxiety or depressive symptoms. There is evidence that certain diet-tracking and fitness apps—particularly those emphasizing calorie counting, weight goals, and quantitative feedback—might inadvertently exacerbate anxiety or disordered eating thoughts in vulnerable users (55). Based on the meta-analytic findings, trials that demonstrated improvements in eating disorder outcomes concurrently exhibited a deterioration in anxiety and depressive symptomatology [(35, 38) see Figure 2]. This pattern suggests that while the intervention effectively targeted the eating disorder construct, it may have inadvertently led to an exacerbation of other related psychological symptoms. One interpretation is that the reduction in eating disorder symptoms could be accompanied by compensatory processes, wherein the focus on one clinical domain shifts or unmasks underlying vulnerabilities in other domains, such as anxiety and depression (56). These findings underscore the complexity inherent in treating multifaceted psychopathologies. Consequently, the meta-analytic results highlight the need for integrated treatment strategies that concurrently address both eating disorder symptoms and comorbid affective disturbances, rather than focusing on a single symptomatic domain (57). Digital interventions should avoid an exclusive emphasis on caloric intake or weight, and instead incorporate elements addressing body image, emotion regulation, and self-compassion to mitigate potential harms (57). It is worth noting that not all research agrees on the risks of dietary self-monitoring. Some studies (58) have found that introducing tracking in a careful, time-limited way does not necessarily worsen mental health. For example, a randomized trial with young adult women (at low eating disorder risk) showed that one month of using a calorie-counting app did not increase eating disorder symptoms, anxiety, or depression, compared to a no-intervention group (38). This suggests that the impact of eating-focused digital tools can vary depending on the population and how the tool is implemented. In low-risk individuals or when paired with protective guidance, self-monitoring might be benign or even helpful for building awareness. However, among adolescents—who often face peer pressure about appearance and may be more impressionable—a purely diet-centric app or intervention could be problematic (59). Thus, the context and design of mHealth eating interventions are crucial. Emphasizing balanced nutrition, mood tracking, and positive psychology (rather than just restricting foods or hitting weight targets) may help ensure that improvements in eating behaviour do not come at the cost of worsened anxiety or mood (60, 61). Integrating mental health support or modules on coping with food-related stress can further safeguard against unintended negative effects (62).

### Depression

Unlike anxiety, which sometimes shows quick wins from behavioural activation, depression may involve an anhedonia and motivational deficit that makes it harder for adolescents to initiate or sustain healthy behaviours without additional support (63). This may explain why depression outcomes improve more gradually and may require multi-component interventions (64). This review suggests that the impact of behavioural mHealth interventions on depression in adolescents may be limited. This aligns with clinical understanding that adolescent depression manifest deeply and requires intensive interventions (65, 66). In our review, improvements in depressive symptoms were small and inconsistent. This is consistent with meta-analytic findings in the literature many digital or behavioural interventions do yield short-term reductions in adolescent depressive symptoms but often rebound (57). For instance, a comprehensive multiverse meta-analysis of digital depression interventions found moderate effect size immediately post-intervention but also noted that effects tended to diminish at follow-ups beyond 24 weeks (67). It is important to acknowledge that some behavioural interventions do confer benefits for depression. Regular physical activity, for example, has been associated with mood improvements in youth (68). A meta-analysis of exercise interventions for adolescents with depression reported a statistically significant antidepressant effect in favour of exercise groups over controls (69). This indicates that structured exercise can indeed reduce depressive symptoms to a meaningful degree, supporting its use as a complementary treatment. In practice, depressive symptoms in adolescents often involve biological, psychological, and social factors that may not be fully remedied by health behaviour change alone.

Coaching, therapy, or even medication might be necessary to augment mHealth programs for depression, ensuring the adolescent stays engaged long enough to benefit.

### Behavioral disorders

For adolescent behavioural disorders our review found that behavioural mHealth interventions had limited effects, but not for the one study on attention-deficit/hyperactivity disorder (ADHD) (44). This is perhaps surprising given that ADHD has a strong neurodevelopmental basis—it is linked to underlying structural and functional brain differences (70) that behavioural interventions alone might not fully overcome (71, 72). That said, there is evidence that structured exercise programs can provide small benefits for youth with ADHD (73), even if they are not a standalone cure. Physical activity has attracted attention as a complementary intervention for ADHD due to its potential effects on executive functioning and self-regulation (74). Some studies in our review (44) noted slight improvements in attention span, mood, or hyperactivity levels following regular exercise in adolescents with ADHD. A systematic review by Neudecker et al. found the largest positive effects from mixed exercise programs (combining aerobic and coordinative activities) on ADHD symptomatology and motor skills (75). However, the findings across studies were highly heterogeneous and often inconsistent. Differences in exercise type, frequency, and study design led to variable outcomes, and the review ultimately concluded that no evidence-based exercise prescription for ADHD could yet be determined (75).

### Strengths and limitations

This review was preregistered and conducted in accordance with PRISMA guidelines and used high quality standards, such as ROB2 assessment and GRADE. Only randomized controlled study designs, including randomized controlled trials, waitlist-controlled trials, quasi-RCTs, were included, and comprehensive literature searches were conducted. A robust assessment of study quality, risk of bias, and heterogeneity using the GRADE framework further bolstered the evaluation. In addition, the systematic application of FITT (Frequency, Intensity, Time, and Type) principles enabled a detailed comparison of intervention characteristics, while the collaboration of a large, multidisciplinary author group enhanced the review's overall robustness. Despite these strengths, the review has three main limitations that warrant caution in interpretation: First, the small number of primary studies (*n* = 9) and the moderate to high heterogeneity observed, particularly for depression and behavioural disorders, limit the generalizability of the findings. The certainty of evidence table underscores these limitations, indicating that the small to moderate pooled effects should be interpreted with caution. Second, high attrition rates, which are well-documented in mHealth studies (76) may inflate the apparent efficacy seen in completers compared to the real-world scenario. For physical activity and sedentary behaviour interventions that address mental health disorders via mHealth interventions, more high-quality RCTs are needed to yield robust meta-analytic conclusions. Expanding our understanding of how mHealth interventions support health behaviours in adolescents is critical for guiding future intervention development and providing ongoing support. Third, an additional limitation of this review is the considerable gender imbalance in the included studies, with female adolescents representing 71% of the total sample. This predominantly female representation, particularly pronounced in studies addressing eating disorders, raises questions about the generalizability of the findings to male adolescents, who may exhibit distinct clinical presentations and respond differently to mHealth interventions. Future research should aim for balanced or gender-specific study designs to better understand gender-specific effects and ensure broader applicability of the findings. Additionally, the relatively short duration and follow-up periods observed in the included studies limit our ability to draw definitive conclusions about the long-term effectiveness of mHealth interventions on mental health outcomes. Given that behavioural and psychological changes may require sustained engagement over time, future research should prioritize long-term follow-up assessments to verify the durability of observed symptom improvements and to identify potential delayed effects or relapses following intervention cessation.

### Implications and directions

The findings suggest that mHealth interventions offer a scalable approach to promote mental health in adolescents, at least for anxiety and eating disorders. Given the widespread access to smartphones, these interventions can complement standard clinical care, particularly mild conditions and in resource-limited settings where traditional face-to-face services are scarce. Although available study data was limited, multicomponent interventions that integrate physical activity, sedentary behaviour, diet, sleep, and are complimented by psychological components appear to offer the most consistent benefits. Enhancing these interventions with interactive features such as gamification (77), self-monitoring, and personalized feedback could further boost adherence and long-term behavioural change (78). Importantly, integrating mHealth tools with standard care, rather than replacing traditional therapies, may optimize outcomes for adolescents with more severe symptoms.

Based on our findings, we recommend mHealth interventions particularly for adolescents with milder symptom presentations, as these groups appear to benefit most from app-based approaches. The effectiveness of interventions increases with frequency and intensity of engagement, indicating that encouraging regular use and adherence could optimize therapeutic outcomes. Therefore, app-based approaches—especially those incorporating hybrid delivery, structured sessions, and multimodal health behaviour targeting—should be prioritized. For practical implementation, we propose a stepped-care approach: initially offering fully digital solutions for milder symptoms and gradually progressing towards hybrid interventions or therapist-supported apps for cases showing greater severity or complexity.

Future research should focus on conducting well-powered, high-quality long term RCTs (preferably with follow up measurements) to investigate the comparative effectiveness of various mHealth intervention components. Research should aim to isolate the impact of features like personalized coaching, peer support, and gamification to determine which strategies best foster engagement and mental health improvements. Given the moderate to high heterogeneity observed in current studies, it is imperative to explore how individual differences (such as developmental stage and baseline mental health status) influence intervention efficacy. Long-term follow-up studies are necessary to assess the durability of improvements in anxiety and eating disorder symptoms, as many digital interventions suffer from high dropout rates. Enhanced strategies to improve sustained engagement—such as adaptive content, push notifications, and social reinforcement—should be a priority. Additionally, future research should consider tailoring mHealth interventions (79) to diverse adolescent populations, addressing factors like gender, socioeconomic status, and cultural context to promote equity in mental health care.

## Conclusion

This review highlights the potential for mHealth interventions to address adolescent mental health, particularly for anxiety and eating disorders. Although digital interventions offer a promising, scalable solution, their effectiveness depends on the integration of multiple lifestyle components and strategies to maintain user engagement. Despite the methodological rigor demonstrated by the predominance of randomized controlled trials, challenges remain with inconsistent reporting of engagement metrics and risk of bias, suggesting that future research should aim to standardize intervention protocols and enhance adherence strategies (80, 81). As highlighted in previous research (15), these trends highlight the potential of integrated, well-supported mHealth interventions tailored to the developmental needs of adolescents (79, 82) while also identifying areas for improvement in future studies. The findings underscore the need for high-quality research to refine intervention strategies, optimize delivery formats, and ensure long-term benefits for adolescents. As the field advances, a balanced approach that combines mHealth with traditional clinical care will likely yield the most sustainable outcomes.

## Data Availability

The original contributions presented in the study are included in the article/Supplementary Material, further inquiries can be directed to the corresponding author.

## References

[1] WHO. Mental health of adolescents. Available at: https://www.who.int/news-room/fact-sheets/detail/adolescent-mental-health (Accessed February 27, 2025).

[2] Polanczyk GV Salum GA Sugaya LS Caye A Rohde LA. Annual research review: a meta-analysis of the worldwide prevalence of mental disorders in children and adolescents. J Child Psychol Psychiatry. (2015) 56(3):345–65. 10.1111/jcpp.12381 25649325

[3] Solmi M Radua J Olivola M Croce E Soardo L Salazar de Pablo G Age at onset of mental disorders worldwide: large-scale meta-analysis of 192 epidemiological studies. Mol Psychiatry. (2022) 27(1):281–95. 10.1038/s41380-021-01161-7 34079068 PMC8960395

[4] Kessler RC Amminger GP Aguilar-Gaxiola S Alonso J Lee S Üstün TB. Age of onset of mental disorders: a review of recent literature. Curr Opin Psychiatry. (2007) 20(4):359–64. 10.1097/YCO.0b013e32816ebc8c 17551351 PMC1925038

[5] Patel C Karasouli E Shuttlewood E Meyer C. Food parenting practices among parents with overweight and obesity: a systematic review. Nutrients. (2018) 10:1966. 10.3390/nu10121966 30545102 PMC6316864

[6] Paus T Keshavan M Giedd JN. Why do many psychiatric disorders emerge during adolescence? Nat Rev Neurosci. (2008) 9(12):947–57. 10.1038/nrn2513 19002191 PMC2762785

[7] Blakemore SJ. Imaging brain development: the adolescent brain. NeuroImage. (2012) 61(2):397–406. 10.1016/j.neuroimage.2011.11.080 22178817

[8] Steinberg L. Cognitive and affective development in adolescence. Trends Cogn Sci. (2005) 9(2):69–74. 10.1016/j.tics.2004.12.005 15668099

[9] Orben A Tomova L Blakemore SJ. The effects of social deprivation on adolescent development and mental health. Lancet Child Adolesc Health. (2020) 4(8):634–40. 10.1016/S2352-4642(20)30186-3 32540024 PMC7292584

[10] Uhlhaas PJ Davey CG Mehta UM Shah J Torous J Allen NB Towards a youth mental health paradigm: a perspective and roadmap. Mol Psychiatry. (2023) 28(8):3171–81. 10.1038/s41380-023-02202-z 37580524 PMC10618105

[11] Gulliver A Griffiths KM Christensen H. Perceived barriers and facilitators to mental health help-seeking in young people: a systematic review. BMC Psychiatry. (2010) 10(1):113. 10.1186/1471-244X-10-113 21192795 PMC3022639

[12] Crone EA Dahl RE. Understanding adolescence as a period of social–affective engagement and goal flexibility. Nat Rev Neurosci. (2012) 13(9):636–50. 10.1038/nrn3313 22903221

[13] Baumann H Meixner C Wollesen B. Voraussetzungen zur vermittlung digitaler gesundheitskompetenzen durch sportlehrkräfte im zuge der SARS-CoV-2-pandemie: eine explorative mixed-methods-studie im schulkontext. Z Stud Lehre Sportwiss. (2022) 5:5–18. 10.25847/zsls.2021.051

[14] Sawyer SM Afifi RA Bearinger LH Blakemore SJ Dick B Ezeh AC Adolescence: a foundation for future health. Lancet Lond Engl. (2012) 379(9826):1630–40. 10.1016/S0140-6736(12)60072-5 22538178

[15] Baumann H. Individualization of mHealth Interventions for Children and Adolescents (Cumulative dissertation to obtain the doctoral degree (Dr. phil.)). Hamburg: University of Hamburg (2023). Available at: https://www.proquest.com/openview/3d34144f44e8a7d532d7f714f89a8bb7/1?pq-origsite=gscholar&cbl=2026366&diss=y (Accessed March 5, 2025).

[16] Kandola A Ashdown-Franks G Hendrikse J Sabiston CM Stubbs B. Physical activity and depression: towards understanding the antidepressant mechanisms of physical activity. Neurosci Biobehav Rev. (2019) 107:525–39. 10.1016/j.neubiorev.2019.09.040 31586447

[17] Firth J Torous J Stubbs B Firth J Steiner G Smith L The ‘online brain’: how the internet may be changing our cognition. World Psychiatry. (2019) 18(2):119–29. 10.1002/wps.20617 31059635 PMC6502424

[18] Khan A Thomas G Karatela S Morawska A Werner-Seidler A. Intense and problematic social media use and sleep difficulties of adolescents in 40 countries. J Adolesc. (2024) 96:1116–25. 10.1002/jad.12321 38570320

[19] Hoare E Collins S Marx W Callaly E Moxham-Smith R Cuijpers P Universal depression prevention: an umbrella review of meta-analyses. J Psychiatr Res. (2021) 144:483–93. 10.1016/j.jpsychires.2021.10.006 34768070

[20] Ricoy MC Martínez-Carrera S Martínez-Carrera I. Social overview of smartphone use by teenagers. Int J Environ Res Public Health. (2022) 19(22):15068. 10.3390/ijerph192215068 36429788 PMC9691203

[21] Free C Phillips G Galli L Watson L Felix L Edwards P The effectiveness of mobile-health technologies to improve health care service delivery processes: a systematic review and meta-analysis. PLOS Med. (2013) 10(1):e1001363. 10.1371/journal.pmed.1001363 23458994 PMC3566926

[22] Singh B Olds T Curtis R Dumuid D Virgara R Watson A Effectiveness of physical activity interventions for improving depression, anxiety and distress: an overview of systematic reviews. Br J Sports Med. (2023) 57(18):1203–9. 10.1136/bjsports-2022-106195 36796860 PMC10579187

[23] O’Neil A Quirk SE Housden S Brennan SL Williams LJ Pasco JA Relationship between diet and mental health in children and adolescents: a systematic review. Am J Public Health. (2014) 104(10):e31–42. 10.2105/AJPH.2014.302110 PMC4167107 25208008

[24] Blacher A McKenzie KNA Stewart SL Reid GJ. Child and adolescent sleep disturbances and psychopathology in a mental health clinic sample. Front Sleep. (2024) 3:1399454. 10.3389/frsle.2024.1399454 PMC12713889 41424509

[25] Zakerabasali S Ayyoubzadeh SM Baniasadi T Yazdani A Abhari S. Mobile health technology and healthcare providers: systemic barriers to adoption. Healthc Inform Res. (2021) 27(4):267–78. 10.4258/hir.2021.27.4.267 34788907 PMC8654335

[26] Zheng S Edney SM Goh CH Tai BC Mair JL Castro O Effectiveness of holistic mobile health interventions on diet, and physical, and mental health outcomes: a systematic review and meta-analysis. eClinicalMedicine. (2023) 66:102309. 10.1016/j.eclinm.2023.102309 38053536 PMC10694579

[27] Fedele DA Cushing CC Fritz A Amaro CM Ortega A. Mobile health interventions for improving health outcomes in youth A meta-analysis. JAMA Pediatr. (2017) 171(5):461–9. 10.1001/jamapediatrics.2017.0042 28319239 PMC6037338

[28] Schardt C Adams MB Owens T Keitz S Fontelo P. Utilization of the PICO framework to improve searching PubMed for clinical questions. BMC Med Inform Decis Mak. (2007) 7(1):16. 10.1186/1472-6947-7-16 17573961 PMC1904193

[29] Veritas Health Innovation. Covidence Systematic Review Software. Melbourna, Australia: Covidence (2025). Available at: www.covidence.org.

[30] Takats S Stillman D. Zotero. Vienna, VA, USA: Corporation for Digital Scholarship (2025).

[31] Higgens J Thomas J Chandler J Cumpston M Li T Page M Cochrane Handbook for Systematic Reviews of Interventions. Version 6.5. Cochrane (2024). Available at: www.training.cochrane.org/handbook. (Accessed February 28, 2025).

[32] Schünemann H Brożek J Guyatt G Oxman A. Handbook for grading the quality of evidence and the strength of recommendations using the GRADE approach. BMJ. (2013) 15. Available at: https://gdt.gradepro.org/app/handbook/handbook.html#h.z014s19g02b2 (Accessed March 5, 2025).

[33] The Cochrane Collaboration. Review Manager (RevMan) (2024). Available at: revman.cochrane.org.

[34] Viechtbauer W. Conducting meta-analyses in R with the metafor package. J Stat Softw. (2010) 36:1–48. 10.18637/jss.v036.i03

[35] Anastasiadou D Folkvord F Brugnera A Cañas Vinader L SerranoTroncoso E Carretero Jardí C An mHealth intervention for the treatment of patients with an eating disorder: a multicenter randomized controlled trial. Int J Eat Disord. (2020) 53(7):1120–31. 10.1002/eat.23286 32383503

[36] Egilsson E Bjarnason R Njardvik U. Usage and daily attrition of a smartphone-based health behavior intervention: randomized controlled trial. JMIR mHealth uHealth. (2023) 11:e45414. 10.2196/45414 37358888 PMC10337294

[37] Fitzsimmons-Craft EE Taylor CB Graham AK Sadeh-Sharvit S Balantekin KN Eichen DM Effectiveness of a digital cognitive behavior therapy-guided self-help intervention for eating disorders in college women: a cluster randomized clinical trial. JAMA Netw Open. (2020) 3(8):e2015633. 10.1001/jamanetworkopen.2020.15633 32865576 PMC7489868

[38] Hahn SL Kaciroti N Eisenberg D Weeks HM Bauer KW Sonneville KR. Introducing dietary self-monitoring to undergraduate women via a calorie counting app has no effect on mental health or health behaviors: results from a randomized controlled trial. J Acad Nutr Diet. (2021) 121(12):2377–88. 10.1016/j.jand.2021.06.311 34427188 PMC9109125

[39] Kauer SD Reid SC Crooke AHD Khor A Hearps SJC Jorm AF Self-monitoring using Mobile phones in the early stages of adolescent depression: randomized controlled trial. J Med Internet Res. (2012) 14(3):e67. 10.2196/jmir.1858 22732135 PMC3414872

[40] Lahtinen O Salmivalli C. An effectiveness study of a digital mindfulness-based program for upper secondary education students. Mindfulness (N Y). (2020) 11(11):2494–505. 10.1007/s12671-020-01462-y

[41] Peuters C Maenhout L Cardon G De Paepe A DeSmet A Lauwerier E A mobile healthy lifestyle intervention to promote mental health in adolescence: a mixed-methods evaluation. BMC Public Health. (2024) 24(1):44. 10.1186/s12889-023-17260-9 38166797 PMC10763383

[42] Werner-Seidler A Li SH Spanos S Johnston L O’Dea B Torok M The effects of a sleep-focused smartphone application on insomnia and depressive symptoms: a randomised controlled trial and mediation analysis. J Child Psychol Psychiatry. (2023) 64(9):1324–35. 10.1111/jcpp.13795 36991537 PMC10952387

[43] Werner-Seidler A Huckvale K Larsen ME Calear AL Maston K Johnston L A trial protocol for the effectiveness of digital interventions for preventing depression in adolescents: the future proofing study. Trials. (2020) 21:2. 10.1186/s13063-019-3901-7 31898512 PMC6941300

[44] Zhao X Lai X Huang S Li Y Dai X Wang H Long-term protective effects of physical activity and self-control on problematic smartphone use in adolescents: a longitudinal mediation analysis. Ment Health Phys Act. (2024) 26:100585. 10.1016/j.mhpa.2024.100585

[45] Granholm A Alhazzani W Møller MH. Use of the GRADE approach in systematic reviews and guidelines. Br J Anaesth. (2019) 123(5):554–9. 10.1016/j.bja.2019.08.015 31558313

[46] Guyatt GH Oxman AD Vist GE Kunz R Falck-Ytter Y Alonso-Coello P GRADE: an emerging consensus on rating quality of evidence and strength of recommendations. Br Med J. (2008 ) 336(7650):924–6. 10.1136/bmj.39489.470347.AD 18436948 PMC2335261

[47] Bevilacqua L Fox-Smith L Lampard O Rojas N Zavitsanou G Meiser-Stedman R Effectiveness of technology-assisted vs face-to-face cognitive behavioural therapy for anxiety and depression in children and young people: a systematic review and meta-analysis. Clin Child Psychol Psychiatry. (2024) 29(4):1349–64. 10.1177/13591045241259070 38870346 PMC13002926

[48] de la Fontaine N Tsafrir S Gothelf D. Rethinking the role of technology-assisted cognitive behavioral therapy for youth in the post-COVID-19 era. Eur Child Adolesc Psychiatry. (2023) 32(4):545–7. 10.1007/s00787-023-02203-x 37022505 PMC10077307

[49] Firth J Solmi M Wootton RE Vancampfort D Schuch FB Hoare E A meta-review of ‘lifestyle psychiatry’: the role of exercise, smoking, diet and sleep in the prevention and treatment of mental disorders. World Psychiatry. (2020) 19(3):360–80. 10.1002/wps.20773 32931092 PMC7491615

[50] Rebar AL Stanton R Geard D Short C Duncan MJ Vandelanotte C. A meta-meta-analysis of the effect of physical activity on depression and anxiety in non-clinical adult populations. Health Psychol Rev. (2015) 9(3):366–78. 10.1080/17437199.2015.1022901 25739893

[51] Parker EL Banfield M Fassnacht DB Hatfield T Kyrios M. Contemporary treatment of anxiety in primary care: a systematic review and meta-analysis of outcomes in countries with universal healthcare. BMC Fam Pract. (2021) 22(1):92. 10.1186/s12875-021-01445-5 33992082 PMC8126070

[52] Aumer T Vögele C. Anxiety reducing effects of physical activity in adolescents and young adults. Eur J Health Psychol. (2025) 32(2):102–19. 10.1027/2512-8442/a000168

[53] Tornberg J Ikäheimo TM Kiviniemi A Pyky R Hautala A Mäntysaari M Physical activity is associated with cardiac autonomic function in adolescent men. PLoS One. (2019) 14(9):e0222121. 10.1371/journal.pone.0222121 31491028 PMC6730886

[54] Bailey AP Castellano G Aleman A. Exercise for the treatment of anxiety in children and adolescents. Cochrane Database Syst Rev. (2022) 9:CD014426. 10.1002/14651858.CD014426

[55] Eikey EV. Effects of diet and fitness apps on eating disorder behaviours: qualitative study. BJPsych Open. (2021) 7(5):e176. 10.1192/bjo.2021.1011

[56] Hartmann S Timm C Barnow S Rubel JA Lalk C Pruessner L. Web-Based cognitive behavioral treatment for bulimia Nervosa: a randomized clinical trial. JAMA Netw Open. (2024) 7(7):e2419019. 10.1001/jamanetworkopen.2024.19019 38958978 PMC11223002

[57] Linardon J Shatte A Messer M Firth J Fuller-Tyszkiewicz M. E-mental health interventions for the treatment and prevention of eating disorders: an updated systematic review and meta-analysis. J Consult Clin Psychol. (2020) 88(11):994–1007. 10.1037/ccp0000575 32852971

[58] Burke LE Wang J Sevick MA. Self-Monitoring in weight loss: a systematic review of the literature. J Am Diet Assoc. (2011) 111(1):92–102. 10.1016/j.jada.2010.10.008 21185970 PMC3268700

[59] Stice E Shaw H Marti CN. A meta-analytic review of eating disorder prevention programs: encouraging findings. Annu Rev Clin Psychol. (2007) 3:207–31. 10.1146/annurev.clinpsy.3.022806.091447 17716054

[60] Hollis C Falconer CJ Martin JL Whittington C Stockton S Glazebrook C Annual research review: digital health interventions for children and young people with mental health problems—a systematic and meta-review. J Child Psychol Psychiatry. (2017) 58(4):474–503. 10.1111/jcpp.12663 27943285

[61] Kristeller JL Wolever RQ. Mindfulness-based eating awareness training for treating binge eating disorder: the conceptual foundation. Eat Disord. (2011) 19(1):49–61. 10.1080/10640266.2011.533605 21181579

[62] Leppanen J Brown D McLinden H Williams S Tchanturia K. The role of emotion regulation in eating disorders: a network meta-analysis approach. Front Psychiatry. (2022) 13:793094. 10.3389/fpsyt.2022.793094 35280172 PMC8904925

[63] Lewinsohn PM Rohde P Seeley JR. Major depressive disorder in older adolescents: prevalence, risk factors, and clinical implications. Clin Psychol Rev. (1998) 18(7):765–94. 10.1016/S0272-7358(98)00010-5 9827321

[64] Weersing VR Brent DA Rozenman MS Gonzalez A Jeffreys M Dickerson JF Brief behavioral therapy for pediatric anxiety and depression in primary care: a randomized clinical trial. JAMA Psychiatry. (2017) 74(6):571–8. 10.1001/jamapsychiatry.2017.0429 28423145 PMC5539834

[65] Merry SN Stasiak K Shepherd M Frampton C Fleming T Lucassen MFG. The effectiveness of SPARX, a computerised self help intervention for adolescents seeking help for depression: randomised controlled non-inferiority trial. Br Med J. (2012) 344:e2598. 10.1136/bmj.e2598 22517917 PMC3330131

[66] Eilert N Wogan R Leen A Richards D. Internet-Delivered interventions for depression and anxiety symptoms in children and young people: systematic review and meta-analysis. JMIR Pediatr Parent. (2022) 5(2):e33551. 10.2196/33551 35551071 PMC9136650

[67] Plessen CY Panagiotopoulou OM Tong L Cuijpers P Karyotaki E. Digital mental health interventions for the treatment of depression: a multiverse meta-analysis. J Affect Disord. (2025) 369:1031–44. 10.1016/j.jad.2024.10.018 39419189

[68] Biddle SJH Asare M. Physical activity and mental health in children and adolescents: a review of reviews. Br J Sports Med. (2011) 45(11):886–95. 10.1136/bjsports-2011-090185 21807669

[69] Oberste M Medele M Javelle F Lioba Wunram H Walter D Bloch W Physical activity for the treatment of adolescent depression: a systematic review and meta-analysis. Front Physiol. (2020) 11:185. 10.3389/fphys.2020.00185 32265725 PMC7096373

[70] Castellanos FX Proal E. Large-scale brain systems in ADHD: beyond the prefrontal-striatal model. Trends Cogn Sci. (2012) 16(1):17–26. 10.1016/j.tics.2011.11.007 22169776 PMC3272832

[71] Yu M Gao X Niu X Zhang M Yang Z Han S Meta-analysis of structural and functional alterations of brain in patients with attention-deficit/hyperactivity disorder. Front Psychiatry. (2023) 13:1070142. 10.3389/fpsyt.2022.1070142 36683981 PMC9853532

[72] Feng A O’Neill S Rostain AL. Contributors to underdiagnosis of ADHD among Asian Americans: a narrative review. J Atten Disord. (2024) 28(12):1499–519. 10.1177/10870547241264113 39082427 PMC11912696

[73] Verret C Guay MC Berthiaume C Gardiner P Béliveau L. A physical activity program improves behavior and cognitive functions in children with ADHD: an exploratory study. J Atten Disord. (2012) 16(1):71–80. 10.1177/1087054710379735 20837978

[74] Pontifex MB Saliba BJ Raine LB Picchietti DL Hillman CH. Exercise improves behavioral, neurocognitive, and scholastic performance in children with ADHD. J Pediatr. (2013) 162(3):543–51. 10.1016/j.jpeds.2012.08.036 23084704 PMC3556380

[75] Neudecker C Mewes N Reimers AK Woll A. Exercise interventions in children and adolescents with ADHD: a systematic review. J Atten Disord. (2019) 23(4):307–24. 10.1177/1087054715584053 25964449

[76] Hightow-Weidman LB Horvath KJ Scott H Hill-Rorie J Bauermeister JA. Engaging youth in mHealth: what works and how can we be sure? mHealth. (2021) 7:23–23. 10.21037/mhealth-20-48 33898592 PMC8063019

[77] Meixner C Baumann H Wollesen B. Personality traits, gamification and features to develop an app to reduce physical inactivity. Information. (2020) 11(7):367. 10.3390/info11070367

[78] Baumann H Fiedler J Wunsch K Woll A Wollesen B. Mhealth interventions to reduce physical inactivity and sedentary behavior in children and adolescents: systematic review and meta-analysis of randomized controlled trials. JMIR MHealth UHealth. (2022) 10(5):e35920. 10.2196/35920 35544294 PMC9133983

[79] Baumann H Heuel L Bischoff LL Wollesen B. Efficacy of individualized sensory-based mHealth interventions to improve distress coping in healthcare professionals: a multi-arm parallel-group randomized controlled trial. Sensors. (2023) 23(4):2322. 10.3390/s23042322 36850920 PMC9963645

[80] He F Qi Y Zhou Y Cao A Yue X Fang S Meta-analysis of the efficacy of digital therapies in children with attention-deficit hyperactivity disorder. Front Psychiatry. (2023) 14:1054831. 10.3389/fpsyt.2023.1054831 37260755 PMC10228751

[81] O’Dean S Sunderland M Newton N Gardner L Teesson M Chapman C The Health4Life e-health intervention for modifying lifestyle risk behaviours of adolescents: secondary outcomes of a cluster randomised controlled trial. Med J Aust. (2024) 220(8):417–24. 10.5694/mja2.52279 38613175

[82] Baumann H Heuel L Bischoff LL Wollesen B. Mhealth interventions to reduce stress in healthcare workers (fitcor): study protocol for a randomized controlled trial. Trials. (2023) 24(1):163. 10.1186/s13063-023-07182-7 36869368 PMC9985281

