# Supplementary material for: Effectiveness of mHealth interventions targeting physical activity, sedentary behaviour, sleep or nutrition on emotional, behavioural and eating disorders in adolescents: a systematic review and meta-analysis
Source: Front Digit Health. 2025 Jul 21;7:1593677. doi: 10.3389/fdgth.2025.1593677 (PMC12318977; doi:10.3389/fdgth.2025.1593677)
Supplement: Supplementary file 1 [file Datasheet1.pdf]

Supplementary Material

Full search strategy:

P(opulation): (adolescent* OR youth OR teenager* OR "young people" OR "young adult*")

I(ntervention): AND ("mHealth" OR "mobile health" OR digital OR "digital intervention*" OR "mobile app*" OR "wearable technolog*" OR "SMS-based intervention*" OR "eHealth" OR "electronic health" OR “Short Message Service” OR SMS OR “Text Messag*” OR “cell phone” OR telephone OR smartphone OR cellular OR mobile) AND ("physical activity" OR exercise OR "sedentary behavior" OR "sedentary lifestyle" OR sleep OR nutrition OR diet OR "health behavior")

C(omprison): (not defined)

O(utcome):AND ("emotional disorder*" OR "mood disorder*" OR anxiety OR "anxiety disorder*" OR "panic disorder" OR "specific phobia" OR "social anxiety disorder" OR "depressive disorder*" OR depression OR dysthymia OR "behavior disorder*" OR ADHD OR "attention deficit disorder" OR "attention deficit hyperactivity disorder" OR "conduct disorder*" OR addiction OR "substance abuse" OR "gaming disorder" OR "eating disorder*" OR "anorexia nervosa" OR "bulimia nervosa" OR "binge eating disorder")
