# Supplementary material for: Effectiveness of mHealth interventions targeting physical activity, sedentary behaviour, sleep or nutrition on emotional, behavioural and eating disorders in adolescents: a systematic review and meta-analysis
Source: Front Digit Health. 2025 Jul 21;7:1593677. doi: 10.3389/fdgth.2025.1593677 (PMC12318977; doi:10.3389/fdgth.2025.1593677)
Supplement: Supplementary file 2 [file Table1.pdf]

Supplementary Table 1. Studies excluded during Full-Text Screening:

Authors	Year	Title	Journal	Reason for Exclusion
**Bannink, R; Broeren, S; Joosten-van Zwanenburg, E; van As, E; van de Looij-Jansen, P; Raat, H**	2014	Effectiveness of a Web-Based Tailored Intervention (E-health4Uth) and Consultation to Promote Adolescents' Health: Randomized Controlled Trial	JOURNAL OF MEDICAL INTERNET RESEARCH 2014;16(5):51-66	Wrong patient population
**Aboody, Dinur; Siev, Jedidiah; Doron, Guy**	2020	Building resilience to body image triggers using brief cognitive training on a mobile application: A randomized controlled trial.	Behav Res Ther 2020;134():103723	Adult population
**Chen, MY; Chia, MC; Chua, T; Shen, Z; Kang, MK; Chen, L; Tong, TT; Wang, XZ**	2024	Associations between Parental Educational Attainment, Children's 24-h Behaviors and Children's Hyperactivity Behavior in the COVID-19 Pandemic	HEALTHCARE 2024;12(5):	Wrong study design
**Schoenfelder, Erin; Moreno, Megan; Wilner, Molly; Whitlock, Kathryn B.; Mendoza, Jason A.**	2017	Piloting a mobile health intervention to increase physical activity for adolescents with ADHD	Preventive Medicine Reports 2017;1():210-213	Wrong study design
**MacLeod, Lucy; Suruliraj, Banuchitra; Gall, Dominik; Bessenyei, Kitti; et al.**	2021	A Mobile Sensing App to Monitor Youth Mental Health: Observational Pilot Study	JMIR MHealth and UHealth 2021;9(10):e20638	Wrong study design
**Tang, CM; Raat, H; Yan, MX; Zhang, Q; Li, KH; Jiang, M; Tang, WJ; Chen, JY; Zhao, Y; Liu, QL**	2021	Application of the health action process approach model for reducing excessive internet use behaviors among rural adolescents in China: a school-based intervention pilot study	BMC PUBLIC HEALTH 2021;21(1):	Wrong intervention
**Chermahini, Melinaz Barati; Eadie, Jazmin; Agarwal, Anika; Stephenson, Callum; Malakouti, Niloufar; Nikjoo, Niloofar; Jagayat, Jasleen; Jarabana, Vineeth; Shirazi, Amirhossein; Kumar, Anchan; et al.**	2024	Comparing the Efficacy of Electronically Delivered Cognitive Behavioral Therapy (e-CBT) to Weekly Online Mental Health Check-Ins for Generalized Anxiety Disorder-A Randomized Controlled Trial: Comparaison de l'efficacité de la thérapie cognitivo-comport	Can J Psychiatry 2024;69(9):695-707	Adult population
**Ashton, L. M.; Morgan, P. J.; Hutchesson, M. J.; Rollo, M. E.; Collins, C. E.**	2017	Feasibility and preliminary efficacy of the 'HEYMAN' healthy lifestyle program for young men: a pilot randomised controlled trial	Nutrition journal 2017;16(1):2	Adult population
**Bress, JN; Falk, A; Schier, MM; Jaywant, A; Moroney, E; Dargis, M; Bennett, SM; Scult, MA; Volpp, KG; Asch, DA; Balachandran, M; Perlis, RH; Lee, FS; Gunning, FM**	2024	Efficacy of a Mobile App-Based Intervention for Young Adults With Anxiety Disorders A Randomized Clinical Trial	JAMA NETWORK OPEN 2024;7(8):	Adult population
**Coringrato, Eva; Alaimo, Katherine; Leiferman, Jenn A.; Villalobos, Angel; Buchenau, Hannah; Decker, Erin; Fahnestock, Lara; Quist, Pallas; Litt, Jill S.**	2024	A process evaluation of a randomized-controlled trial of community gardening to improve health behaviors and reduce stress and anxiety	Sci Rep 2024;14(1):13620	Wrong intervention
**Dang, Kevin; Ritvo, Paul; Katz, Joel; Gratzer, David; Knyahnytska, Yuliya; Ortiz, Abigail; Walters, Clarice; Attia, Mohamed; Gonzalez-Torres, Christina; Lustig, Andrew; Daskalakis, Zafiris**	2023	The Role of Daily Steps in the Treatment of Major Depressive Disorder: Secondary Analysis of a Randomized Controlled Trial of a 6-Month Internet-Based, Mindfulness-Based Cognitive Behavioral Therapy Intervention for Youth	Interactive Journal of Medical Research 2023;1():e46419	Adult population
**Eikey, EV; Reddy, MC; Booth, KM; Kvasny, L; Blair, JL; Li, V; Poole, ES**	2017	Desire to Be Underweight: Exploratory Study on a Weight Loss App Community and User Perceptions of the Impact on Disordered Eating Behaviors	JMIR MHEALTH AND UHEALTH 2017;5(10):	Wrong study design
**Fitzsimmons-Craft, Ellen E.; Firebaugh, Marie-Laure; Graham, Andrea K.; et al.**	2019	State-wide university implementation of an online platform for eating disorders screening and intervention	Psychological Services 2019;16(2):239-249	Wrong study design

**Aslund, Li; Jernelov, Susanna; Serlachius, Eva; Vigerland, Sarah; Wicksell, Rikard K.; Henje, Eva; Lekander, Mats**	2024	Internet-delivered cognitive behavioral therapy for adolescents with insomnia: Feasibility and preliminary efficacy	Clinical Child Psychology & Psychiatry 2024;29(3):1159-1173	Wrong study design
**Hahn, Samantha L.; Hazzard, Vivienne M.; Loth, Katie A.; Larson, Nicole; Klein, Laura; Neumark-Sztainer, Dianne**	2022	Using apps to self-monitor diet and physical activity is linked to greater use of disordered eating behaviors among emerging adults	Preventive Medicine: An International Journal Devoted to Practice and Theory 2022;():1-7	Wrong study design
**Pitrat, B.; Turpin, A.; Peyret, E.; Hamonniere, T.; Drain, A.; Maatoug, R.; Le Roux, E.**	2024	Mobile app measuring sleep and behaviors: a trial in adolescents with addiction	Encephale 2024;()	Wrong study design
**Haug, Severin; Paz Castro, Raquel; Scholz, Urte; Kowatsch, Tobias; Schaub, Michael Patrick; Radtke, Theda**	2020	Assessment of the Efficacy of a Mobile Phone-Delivered Just-in-Time Planning Intervention to Reduce Alcohol Use in Adolescents: Randomized Controlled Crossover Trial.	JMIR Mhealth Uhealth 2020;8(5):e16937	No PA/SB/Nutrition/Sleep
**Babic, M. J.; Smith, J. J.; Morgan, P. J.; Lonsdale, C.; Plotnikoff, R. C.; Eather, N.; Skinner, G.; Baker, A. L.; Pollock, E.; Lubans, D. R.**	2016	Intervention to reduce recreational screen-time in adolescents: outcomes and mediators from the 'Switch-Off 4 Healthy Minds' (S4HM) cluster randomized controlled trial	Preventive medicine 2016;91():50-57	Wrong intervention
**Chen, S. J.; Que, J. Y.; Chan, N. Y.; Li, S. X.; Zhang, J. H.; Zhong, Y.; Ho, Y. L.; Tsang, C. C.; Shi, L.; Lu, L.; et al.**	2024	Effectiveness of e-based cognitive behavioral therapy for insomnia on enhancing depression and insomnia outcome in Chinese youth with both diagnoses	Sleep medicine 2024;115():171	Wrong intervention
**Wiljer, D.; Shi, J.; Lo, B.; Sanches, M.; Hollenberg, E.; Johnson, A.; Abi-Jaoudé, A.; Chaim, G.; Cleverley, K.; Henderson, J.; et al.**	2020	Effects of a Mobile and Web App (Thought Spot) on Mental Health Help-Seeking Among College and University Students: randomized Controlled Trial	Journal of medical Internet research 2020;22(10):e20790	Wrong patient population
**Adikari, Amgcp; Appukutty, M.; Kuan, G.**	2020	Effects of Daily Probiotics Supplementation on Anxiety Induced Physiological Parameters among Competitive Football Players	Nutrients 2020;12(7):	Wrong intervention
**Whitehouse, Sandy R; Lam, Pei-Yoong; Balka, Ellen; McLellan, Shelagh; Deevska, Mariana; Penn, Daniel; Issenman, Robert; Paone, Mary**	2013	Co-Creation With TickiT: Designing and Evaluating a Clinical eHealth Platform for Youth	JMIR Res Protoc 2013;2(2):e42	Wrong study design
**Pham, Q; Khatib, Y; Stansfeld, S; Fox, S; Green, T**	2016	Feasibility and Efficacy of an mHealth Game for Managing Anxiety: "Flowy" Randomized Controlled Pilot Trial and Design Evaluation	GAMES FOR HEALTH JOURNAL 2016;5(1):50-67	Adult population
**Braddock, Amy; Ghosh, Parijat; Montgomery, Emma; Lim, Crystal; Ghosh, Jaya; Henry, Nicole; Popescu, Mihail; et al.**	2024	Effectiveness of an mHealth App That Uses Financial Incentives and Gamification to Promote Health Behavior Change in Adolescents and Caregivers: Protocol for a Clinic-Based Randomized Controlled Trial	JMIR Res Protoc 2024;13():e63505	Wrong study design
**Dietvorst, E.; Legerstee, J. S.; Vreeker, A.; Koval, S.; Mens, M. M.; Keijsers, L.; Hillegers, M. H. J.**	2023	The Grow It! app—longitudinal changes in adolescent well-being during the COVID-19 pandemic: a proof-of-concept study	Eur Child Adolesc Psychiatry 2023;32(6):1097-1107	Wrong study design
**Chen, Robert Yuzen; Feltes, Jordan Robert; Tzeng, William Shun; Lu, Zoe Yunzhu; Pan, Michael; Zhao, Nan; Talkin, Rebecca; Javaherian, Kavon; Glowinski, Anne; Ross, Will**	2017	Phone-Based Interventions in Adolescent Psychiatry: A Perspective and Proof of Concept Pilot Study With a Focus on Depression and Autism	JMIR Research Protocols 2017;6(6):e114	Wrong study design
**Imani, V.; Pakpour, A.**	2024	An online sleep intervention for adolescents who are gaming	Sleep medicine 2024;115():159-160	Wrong study design
**Hung, Shan; Li, Min-Shan; Chen, Yen-Lin; Chiang, Jung-Hsien; Chen, Ying-Yeh; Hung, Galen Chin-Lun**	2016	Smartphone-based ecological momentary assessment for Chinese patients with depression: An exploratory study in Taiwan	Asian Journal of Psychiatry 2016;1():131-136	Wrong study design

**Raevuori, Anu; Vahlberg, Tero; Korhonen, Tellervo; Hilgert, Outi; Aittakumpu-Hyden, Raija; Forman-Hoffman, Valerie**	2021	A therapist-guided smartphone app for major depression in young adults: A randomized clinical trial.	J Affect Disord 2021;286():228-238	Wrong intervention
**Hilt, L. M.; Swords, C. M.; Webb, C. A.**	2023	Randomized Controlled Trial of a Mindfulness Mobile Application for Ruminative Adolescents	Journal of clinical child and adolescent psychology 2023;():1-14	Wrong outcomes
**Leigh, Eleanor; Clark, David M.**	2023	Internet-delivered therapist-assisted cognitive therapy for adolescent social anxiety disorder (OSCA): a randomised controlled trial addressing preliminary efficacy and mechanisms of action.	J Child Psychol Psychiatry 2023;64(1):145-155	Wrong intervention
**Pramana, Gede; Parmanto, Bambang; Kendall, Philip C.; Silk, Jennifer S.**	2014	The SmartCAT: An m-Health Platform for Ecological Momentary Intervention in Child Anxiety Treatment	Telemedicine and e-Health 2014;20(5):419-427	Wrong study design
**Perez-Vazquez, J.; Gonzalez-Roz, A.; Amigo-Vazquez, I.**	2024	Effectiveness of an e-Health Quasi-Randomized Controlled Universal Prevention Program for Eating Disorders in Spanish Adolescents	Journal of prevention (2022) 2024;45(1):87-105	Wrong study design
**Boucher, EM; Ward, H; Miles, CJ; Henry, R; Stoeckl, SE**	2024	Effects of a Digital Mental Health Intervention on Perceived Stress and Rumination in Adolescents Aged 13 to 17 Years: Randomized Controlled Trial	JOURNAL OF MEDICAL INTERNET RESEARCH 2024;26():	Wrong outcomes
**Gex, Kathryn S.; Mun, Eun-Young; Barnett, Nancy P.; McDevitt-Murphy, Meghan E.; Ruggiero, Kenneth J.; Thurston, Idia B.; Olin, Cecilia C.; Voss, Andrew T.; Withers, Alton J.; Murphy, James G.**	2023	A randomized pilot trial of a mobile delivered brief motivational interviewing and behavioral economic alcohol intervention for emerging adults.	Psychol Addict Behav 2023;37(3):462-474	Wrong patient population
**Nct, –**	2024	i-TREAT: an Internet-based Treatment for Eating Disorders	(ClinicalTrials.gov link provided)	Wrong study design
**Lawrence-Sidebottom, Darian; Huffman, Landry Goodgame; Beam, Aislinn; Parikh, Amit; Guerra, Rachael; Roots, Monika; Huberty, Jennifer**	2024	Improvements in sleep problems and their associations with mental health symptoms: A study of children and adolescents participating in a digital mental health intervention	Digital Health 2024;1():20552076241249928	Wrong study design
**De Cock, Nathalie; Van Lippevelde, Wendy; Vangeel, Jolien; Notebaert, Melissa; et al.**	2018	Feasibility and impact study of a reward-based mobile application to improve adolescents' snacking habits	Public Health Nutr. 2018;21(12):2329-2344	Wrong study design
**Farrer, L. M.; Jackson, H. M.; Gulliver, A.; Calear, A. L.; Leach, L.; Hasking, P.; Katruss, N.; Batterham, P. J.**	2024	A Transdiagnostic Video-Based Internet Intervention (Uni Virtual Clinic-Lite) to Improve the Mental Health of University Students: randomized Controlled Trial	Journal of medical Internet research 2024;26():e53598	Adult population
**Willems, L; Rasing, SPA; Heijs, DAM; Vermulst, AA; Huvenaars, MJ; Onrust, SA; Creemers, DHM**	2024	Mental health app boost my mood (BMM) as preventive early intervention for adolescents with (sub)clinical depressive symptoms	BMC PUBLIC HEALTH 2024;24(1):	Wrong study design
**Pinto, Melissa D.; Greenblatt, Amy M.; Hickman, Ronald L.; Rice, Heather M.; Thomas, Tami L.; Clochesy, John M.**	2016	Assessing the critical parameters of eSMART-MH: A promising avatar-based digital therapeutic intervention to reduce depressive symptoms	Perspectives in Psychiatric Care 2016;52(3):157-168	Adult population
**Ko, K. S.; Lee, W. K.**	2023	A preliminary study using a mobile app as a dance/movement therapy intervention to reduce anxiety and enhance the mindfulness of adolescents in South Korea	Arts in psychotherapy 2023;85():	No PA/SB/Nutrition/Sleep
**Palmer, Melanie; Beckley-Hoelscher, Nicholas; Shearer, James; Kostyrka-Allchorne, Katarzyna; Robertson, Olly; Koch, Marta; Pearson, Oliver; et al**	2023	The Effectiveness and Cost-Effectiveness of a Universal Digital Parenting Intervention Designed and Implemented During the COVID-19 Pandemic: Evidence From a Rapid-Implementation Randomized Controlled Trial Within a Cohort.	J Med Internet Res 2023;25():e44079	Wrong patient population

**Timpel, P.; Cesena, F. H. Y.; da Silva Costa, C.; Soldatelli, M. D.; Gois, E.; Castrillon, E.; Diaz, L. J. J.; Repetto, G. M.; Hagos, F.; Castillo Yermenos, R. E.; et al.**	2018	Efficacy of gamification-based smartphone application for weight loss in overweight and obese adolescents: study protocol for a phase II randomized controlled trial	Therapeutic advances in endocrinology and metabolism 2018;9(6):167-176	Wrong study design
**Ahmad, F.; El Morr, C.; Ritvo, P.; Othman, N.; Moineddin, R.**	2020	An eight-week, web-based mindfulness virtual community intervention for students' mental health: randomized controlled trial	JMIR mental health 2020;7(2):e15520	Adult population
**van Aubel, Evelyne; Bakker, Jindra Myrthe; Batink, Tim; Michielse, Stijn; Goossens, Liesbet; Lange, Iris; Schruers, Koen; Lieverse, Ritsaert; Marcelis, Machteld; van Amelsvoort, Thérèse; van Os, Jim; Wichers, Marieke; Vaessen, Thomas; Reininghaus, Ulrich; Myin-Germeys, Inez**	2020	Blended care in the treatment of subthreshold symptoms of depression and psychosis in emerging adults: A randomised controlled trial of Acceptance and Commitment Therapy in Daily-Life (ACT-DL).	Behav Res Ther 2020;128():103592	Wrong intervention
**Hilt, LM; Swords, CM; Austria, N; Webb, CA; Wahl, J; Eklund, L**	2024	Nonjudgment Mediates the Effect of a Brief Smartphone-Delivered Mindfulness Intervention on Rumination in a Randomized Controlled Trial with Adolescents	MINDFULNESS 2024;15(5):1208-1219	Wrong intervention
